# How to awaken a sleeping giant: antagonistic expression of Flowering locus T homologs and elements of the age-related pathway are associated with the flowering transition in *Agave tequilana*

**DOI:** 10.1007/s00497-023-00489-0

**Published:** 2023-12-12

**Authors:** Laura Hernández-Soriano, Laura Gálvez-Sandre, Emmanuel Ávila de Dios, June Simpson

**Affiliations:** 1 Department of Genetic Engineering, Cinvestav Unidad Irapuato, Guanajuato, Mexico; 2 Present Address: Department of Botany and Plant Sciences, University of California, Riverside, CA 92521 USA

**Keywords:** *Agave tequilana*, Flowering locus T, Apetala 2, Squamosa Promoter Binding-Like, miRNAs, PGHF32

## Abstract

**Key message:**

Antagonistic expression of Flowering locus T proteins and the ageing pathway via miRNAs and sugar metabolism regulate the initiation of flowering in *A. tequilana*.

**Abstract:**

Flowering in commercial plantations of *Agave tequilana* signals that plants are ready to harvest for tequila production. However, time of flowering is often unpredictable and a detailed understanding of the process would be beneficial in the field, for breeding and for the development of future research. This report describes the functional analysis of *A. tequilana* FLOWERING LOCUS T (FT) genes by heterologous expression in *A. thaliana* and in situ hybridization in agave plants. The gene structures of the *Agave tequilana* FT family are also described and putative regulatory promoter elements were identified. Most *Agave* species have monocarpic, perennial life cycles that can last over 25 years during which plants do not respond to the normal environmental signals which induce flowering, suggesting that the ageing pathway as described in Arabidopsis may play an important role in determining flowering time in these species. Elements of this pathway were analyzed and in silico data is presented that supports the regulation of SQUAMOSA PROMOTER BINDING LIKE proteins (SPL), APETALA2 (AP2) proteins and members of Plant Glycoside Hydrolase Family 32 (PGHF32) by interactions with miRNAs 156, 172 and 164 during the initiation of flowering in *A. tequilana*.

**Supplementary Information:**

The online version contains supplementary material available at 10.1007/s00497-023-00489-0.

## Introduction

*A. tequilana* is an important crop species in Mexico, due to its capacity for synthesis and storage of fructans, which accumulate in a structure known as the "piña", made up of the stem and basal leaf tissue (Lopez et al. 2003). Fructans constitute the essential raw material to produce tequila and mezcal (iconic beverages of Mexico, Valenzuela-Sánchez et al. 2008) and although tequila production must be based on *A. tequilana* (NOM-006-SCFI-2012 (2012)), mezcal, can be produced from different individual species or even mixtures of different species. In addition, the *Agave* genus has attracted interest for applications in the food industry as substitutes for sugars, for the extraction of bioactive compounds, and in bioethanol production (Bautista-Montes et al. 2022).

Most *Agave* species are monocarpic perennials with life cycles between 5 and at least 25 years. The inflorescence of agave plants can reach more than 10 m in height, growing at around 10 cm per day and is the largest inflorescence of any plant species (Gentry 1982). *A. tequilana* plants flower between 5 and 7 years after planting and can produce > 16,000 flowers per plant and > 82,000 seeds (Escobar-Guzman et al. 2008).

Commercial plantations are closely monitored in order to remove the inflorescences and suppress the flowering process as soon as is feasible in order to conserve the carbohydrates accumulated in the stem. Flowering within a single plantation however is heterogeneous with individual plants flowering in successive years. Agave producers must therefore decide whether to harvest the same plantation in successive years assuring optimum levels of carbohydrates or harvest the plantation in a single year and include plants below optimum (Ávila de Dios et al. 2019). The ability to predict the onset of the vegetative to reproductive transition in *A. tequilana* and/or suppress flowering indefinitely would be a great advantage for commercial production by eliminating the need for the removal of the inflorescence and allowing harvesting throughout the year. In contrast, the possibility to accelerate flowering would make *Agave* species more amenable to classical breeding strategies.

The initiation of the reproductive stage in the life cycle of flowering plants is a crucial event (Casal et al. 2004) and the vegetative to reproductive transition in annual plants usually leads to irreversible changes in developmental patterns. Because of this, plants have adopted various mechanisms to synchronize flowering with favorable environmental conditions (Bergonzi and Albani 2011), employing a complex regulatory network that integrates endogenous and environmental signals (Amasino 2010).

In *Arabidopsis thaliana*, an annual plant with a short life cycle, the reproductive transition is regulated by five main pathways associated with environmental or endogenous factors and denominated: the photoperiodic pathway, the vernalization pathway, the autonomous pathway, the gibberellin pathway and the ageing pathway (Teotia & Tang 2015). These pathways converge in a pivotal protein denominated Flowering locus T (FT) (Lifschitz 2014) belonging to the phosphatidylethanolamine-binding protein family (Kobayashi et al. 1999). *ft-10* an *Agrobacterium tumefasciens* insertion mutant of *A. thaliana* FT (AtFT) with very low levels of FT expression shows a very late flowering phenotype where plants have around 40 leaves in contrast to 15 leaves in wild type plants at the flowering stage (Yoo et al. 2005). Overexpression of CONSTANS (CO), the main regulator of the photoperiod pathway, leads to an early flowering phenotype. However, overexpression of CONSTANS in combination with the *ft-10* mutant led to a late flowering phenotype, indicating that CO targets FT to promote flowering.

Members of the phosphatidylethanolamine-binding protein (FT/TFL) family have been studied in *A. thaliana* and include: FLOWERING LOCUS T (FT), TERMINAL FLOWER1 (TFL1), TWIN SISTER OF FT (TSF), BROTHER OF FT (BFT), ARABIDOPSIS THALIANA CENTRORADIALIS HOMOLOG (ATC) and MOTHER OF FT (MFT) (Kobayashi et al.^1999^; Yoo et al. 2004) (Michaels 2009) (Yamaguchi et al. 2005). The TSF paralog is the closest homolog to FT, in terms of protein sequence, diurnal expression pattern and flowering induction when overexpressed (Yamaguchi et al. 2005). BFT also shows high sequence similarity and daytime expression with FT, but functions as a repressor similar to TFL1 (Yoo et al. 2004). ATC suppresses short photoperiods and it has been suggested that this protein in common with FT may be transported from the vasculature to the SAM (Lu et al. 2012). MFT shares a strong homology with FT and TFL1 and also participates in floral induction possibly acting redundantly with FT. It has been suggested that it is an ancestral form of FT and TFL1, since basal plant species such as mosses have MFT homologs, but not FT or TFL1 (Karlgren et al. 2011).

Although FT genes are expressed in the phloem companion cells in leaves (Corbesier et al. 2007), FT proteins are transported via the phloem to the Shoot Apical Meristem (SAM). In the SAM, FT binds to the transcription factor FD and the 14–3–3 proteins creating the florigen-activation complex (FAC) and triggering the expression of meristem identity genes such as APETALA1 (AP1) and FRUITFULL (FUL) (Abe et al. 2005; Wigge et al. 2005; Taoka et al. 2011).

Inducer and repressor FT homologs have previously been characterized in annual monocarpic and perennial polycarpic species in relation to flowering (Winterhagen et al. 2013) and in biennial crops such as onion (*A. cepa*) and sugar beet (*B. vulgaris*) in relation to bulbing and flowering, respectively (Lee et al. 2013; Pin et al. 2010). Although some reports are available for bamboo species (Guo et al. 2016; Yang et al. 2019) the characterization of FT is limited in other perennial monocarpic species such as *Agave tequilana*. Transcriptome analysis of leaves and shoot apical meristem tissues of *A. tequilana* plants from different developmental stages, revealed a series of factors involved in the reproductive transition including MADS-box transcription factors, gibberellin signaling, carbohydrate metabolism-related genes and genes of the Flowering locus T family (AtqFT) (Ávila De Dios et al. 2019). The putative functions of the AtqFTs were determined based on phylogenetic analyses and the presence of key amino acid residues. Predicted functions were also supported by in silico expression analysis (Ávila De Dios et al. 2019). Based on the results obtained, *AtqFT1*, *AtqFT2*, *AtqFT8*, *AtqFT9* and *AtqFT10* are predicted to be promoters of flowering, whereas *AtqFT4* and *AtqFT11* are predicted to be repressors.

FT proteins have been proposed to integrate responses to elements of the different pathways that lead to the reproductive transition and given the perennial monocarpic life cycle of *Agave* species it is plausible to predict that the ageing pathway involving miRNAs and sugar signaling plays an important role in this process. Hexokinase signaling has been linked to the regulation of expression of miR156 (Granot et al. 2014), a negative regulator of some members of the SQUAMOSA PROMOTER BINDING LIKE (SPL) transcription factor family that control the expression of miR172. miR172 on the other hand represses genes of the APETALA2/ETHYLENE RESPONSIVE FAMILY (AP2/ERF) that inhibit the initiation of the transition to the reproductive stage (Aukerman and Sakai 2003; Schmid et al. 2003; Chen 2004).

Ren et al. (2022) have shown the importance of miRNA-mRNA networks associated with carbohydrate metabolism during bud abortion in lotus (*Melumbo nucifera*) induced by low-light conditions and Ávila De Dios et al. (2019) reported that genes from plant glycoside hydrolase family 32 (PGHF32) which contains invertases (cell wall and vacuolar), fructan exohydrolases and fructosyltransferases are differentially regulated during the vegetative to reproductive transition in *A. tequilana.* Gomez-Vargas et al. (2022) also demonstrated that several PGHF32 genes are also light regulated in *A. tequilana* leaf tissue supporting a putative role for the ageing pathway and carbohydrate metabolism in the reproductive transition.

This report describes the functional characterization of the *AtqFT1*, *AtqFT2*, and *AtqFT4* genes of *A. tequilana* in the heterologous system of *A. thaliana* during the process of the transition to the reproductive phase and shows their specific expression in leaf vascular tissue. The gene structure and putative regulatory motifs of the *A. tequilana* FT genes are also reported for the first time. Additionally, in silico data describing the presence of miRNA target sequences in *A. tequilana* AP2, SPL and PGHF32 gene families putatively involved in the vegetative to reproductive transition and the expression patterns of selected miRNAs and their target genes is presented.

## Materials and methods

### Gene characterization

In order to obtain the *AtqFT* genomic sequences, the predicted cDNA sequences for AtqFT1 (MK251781), AtqFT2 (MK251782), AtqFT3 (MK251783), AtqFT4 (MK251784), AtqFT5 (MK251785), AtqFT6 (MK251786), AtqFT8 (MK251788), AtqFT9 (MK251789), and AtqFT10 (MK251790) (Ávila De Dios et al. 2019) were compared to a draft genome of *Agave tequilana* (Herrera-Estrella et al. unpublished) using the tBLASTn + 2.12.0 algorithm. Access to the Cinvestav *A. tequilana* genome database can be obtained by contacting Alfredo Herrera-Estrella (alfredo.herrera@cinvestav.mx). Transcriptome sequences are available at https://www.ncbi.nlm.nih.gov/genbank (accessed on 18 Jan 2023). The gene structures of the AtqFTs were determined by alignments of transcriptomic and genomic sequences using the Muscle algorithm in MEGA software (Sudhir et al. 1993), version 10.2.5 https://www.megasoftware.net/ (accessed on 18 Jan 2023). Genomic sequences are registered in GenBank under accession numbers: AtqFT1 OR887596, AtqFT2 OR887597, AtqFT3 OR887598, AtqFT4 OR887599, AtqFT5 OR887600, AtqFT8 OR887601, AtqFT9 OR887602, AtqFT10 OR887603.

### Determination of Cis Motifs in *AtqFT* promoters

To identify regulatory motifs in the AtqFT promoter regions, a 2 kb region upstream the transcription start site was analyzed using the “*Plant cis-acting regulatory DNA elements”* (New PLACE), database https://www.dna.affrc.go.jp/PLACE/?action=newplace (accessed on 18/01/2023).

### Plant material

*Agave tequilana* plants were sampled in Irapuato Guanajuato, Mexico (20.717483, − 101.365117) in February 2021. Shoot apical meristems and leaf tissue at the vegetative and early reproductive stages of the reproductive transition (Delgado Sandoval et al. 2012) were collected and used to obtain complete cDNAs of AtqFT1, 2 and 4. *Arabidopsis thaliana* (Columbia-0) wild type and the *ft-10* mutant background were used for heterologous expression of *A. tequilana* FT cDNAs.

### Gene isolation and cDNA cloning

Leaf and shoot apical meristem tissues of *A. tequilana* plants were collected in duplicate at the vegetative and early reproductive developmental stages, frozen in liquid nitrogen and stored at − 80 °C until needed. Tissues were ground to a powder using a Tissue Lyser (QIAGEN) and RNA was isolated using the TRizol reagent (Invitrogen) in combination with the PureLink RNA Mini Kit Purification System (Invitrogen) according to the manufacturer’s instructions.

RT-PCR was carried out using an oligo dt primer, 1 μg RNA and SuperScript II Reverse Transcriptase (Invitrogen) according to the manufacturer’s instructions. To obtain the full length AtqFTs, primers were designed for each homolog (Online Resource 1) and a RT-PCR was carried out under the following conditions: 1 cycle at 95 °C for 5 min; 25 cycles at 95 °C for 0.5 min, 58 °C for 0.5 min, and 72 °C for 0.5 min; 1 cycle at 72 °C for 7 min. PCR products were cloned using the Gateway system into the entry vector pDONR222 and the expression vector pB7WG2D under the control of the cauliflower mosaic virus (CaMV) 35S promoter and transferred to *Agrobacterium tumefaciens* GV2260.

### Plant transformation

*Arabidopsis thaliana* plants were transformed with the floral dip method Martinez-Trujillo et al. (2004). The inoculum was applied using a micropipette to closed flower buds for four weeks. The independently obtained lines for each construction were chosen after selection on 4 mg/L glufosinate and according to phenotype and PCR analysis.

### In situ hybridization

Transverse sections of leaves and longitudinal sections of shoot apical meristem (SAM) tissues from each developmental stage were dissected and fixed in FAA (3.7% formaldehyde, 50% ethanol, and 5% acetic acid). Tissues were dehydrated through an ethanol series and embedded in paraffin. Paraffin blocks were sectioned using a microtome. In situ hybridization was performed according to Ruiz-Medrano et al. (1999) and Abraham-Juárez et al. (2010). Riboprobes were synthetized using the SP6/T7 in vitro transcription kit (Promega) and UTP-digoxigenin (Roche) as the label. Detection was performed using NBT/BCIP (Roche) and images were obtained using an Olympus BX60 microscope fitted with an Olympus DP71 camera.

### Analysis of AtqFT genomic sequences

To obtain the genomic sequences, a tBLASTn analysis was performed against the *A. tequilana* genome version 3.1 using the transcriptome sequences reported by Ávila de Dios et al. (2019). The best hits were obtained by selecting those with the smaller the E-value and the higher bit-score. The genome coordinates were also determined for each cDNA. Genomic and cDNA sequences were compared in order to predict the putative exon/intron structure of the AtqFTs based on alignments using the Muscle algorithm.

To identify putative regulatory elements in the AtqFT promoter regions 2 kb regions upstream the transcription factor start site were analyzed using the “New PLACE” database (https://www.dna.affrc.go.jp/PLACE/?action=newplace).

### Identification of *A. tequilana* AP2 and SPL gene families

In the transcriptome generated by Ávila de Dios et al. (2019) based on samples from leaf and shoot apical meristem tissue sampled from 4 year old field grown agave plants (vegetative phase samples) and 6 year old field grown agave plants in the initial stage of the flowering transition (reproductive phase) with 3 biological replicates, a search was made for annotated sequences using the keywords "SQUAMOSA", "SQUAMOSA PROMOTER BINDING LIKE", "SBP", "SPL", "AP2” “AP2/ERF” “RAP2″, “APETALA2-LIKE”. The sequences obtained were downloaded in FASTA format and imported into the Geneious 4.8.5 platform for subsequent analysis. AP2 and SPL cDNA sequences are deposited in GenBank under accession numbers:Atq_AP2 OR751380, Atq_AP2-2 OR751381, Atq_ANT OR751382, Atq_SPL1 OR751383, Atq_SPL2 OR751384, Atq_SPL3 OR751385, Atq_SPL4 OR751386, Atq_SPL5 OR751387, Atq_SPL6 OR751388, Atq_SPL7 OR751389, Atq_SPL8 OR751390, Atq_SPL9 OR751391, Atq_SPL10 OR751392, Atq_SPL11 OR751393, Atq_SPL12 OR751394, Atq_SPL13 OR751395.

### Identification of putative transcription factor sequences

Sequences obtained from the transcriptome were manually filtered to remove incomplete or incorrectly annotated sequences. Only sequences with the complete open reading frame were considered, and they were reviewed manually and with the NCBI tblastn tool. Reading frames were extracted and ordered using the EMBL-EBI MUSCLE (Multiple Sequence Comparison by Log-Expectation) tool within the Geneious program.

### Construction of dendrograms of the AP2 and SPL families

Selected sequences of each family were aligned with the MUSCLE suite, then imported into the MEGA-X program (Molecular Evolutionary Genetics Analysis). The dendrograms were produced using the maximum likelihood method and a Bootstrap of 5000.

### Determination of expression levels

In silico expression data generated previously (Ávila de Dios et al. 2019) was filtered for each of the identified transcripts assigned to a cluster. Expression levels were represented as transcripts per million (TPM).

### Identification of conserved miRNAs

Small RNAs were extracted from leaf and shoot apical meristem tissue sampled from three 4 year old field grown agave plants (vegetative phase samples) and three 6 year old field grown agave plants in the initial stage of the flowering transition (reproductive phase). 100 mg of tissue from each tissue and each developmental stage were taken from each plant and ground in liquid nitrogen. The *mir*Vana miRNA isolation kit (Thermo Fisher Scientific) was used for the extraction protocol. cDNAs generated from the extracted RNA were sequenced on the Illumina Nextseq 500 platform at the Advanced Genomics Unit at Cinvestav, Irapuato. Sequences were filtered to remove those without 3′ adapters and adapters were subsequently removed from the remaining sequences. 5′ adapters were removed using Cutadapt and sequences less than 18 nucleotides in length were discarded. Low quality sequences were also eliminated. Since no genome data was available when the agave small RNAs were prepared, sequences were compared against non-coding RNA sequences from different plant species classified as distinct from miRNAs using the criteria that no errors in alignment were allowed. Only the sequences that did not align with these databases were accepted as candidate miRNAs and used in subsequent analyses.

To identify conserved miRNAs, the criteria used was to consider those that were reported in at least one plant in the miRBase database (http://www.mirbase.org/) based on 20–25 nucleotide reads identified in the *A. tequilana* miRNA database (data not shown). miRNA sequences were aligned using the Bowtie program allowing up to two errors in the alignment.

### miRNA prediction

The readings that did not align to conserved miRNAs were used to predict putative miRNAs, using the miR-PREFeR program (Lei and Sun 2014). Briefly, the reads for each library were aligned against the transcriptome database allowing for 1 error in the alignment, the files in SAM (Sequence Alignment Map) format and the transcriptome were fed into miR-PREFeR, which identified regions of up to 300 nucleotides in length that could form a stem-loop structure and for which there are readings that support the miRNA and its miRNA*. miRNA data is available in GenBank with the BioProject ID: PRJNA1048505.

### Target gene prediction of conserved and predicted miRNAs

The prediction of the target genes of the miRNAs, both conserved and predicted, was based on analysis of the transcriptome database. The prediction was made through the psRNATarget site (http://plantgrn.noble.org/psRNATarget) using the configuration called “V2 2017” (Dai et al. 2018).

## Results

### Timing of the flowering process in *Agave* species shows plasticity

**Fig. 1:**
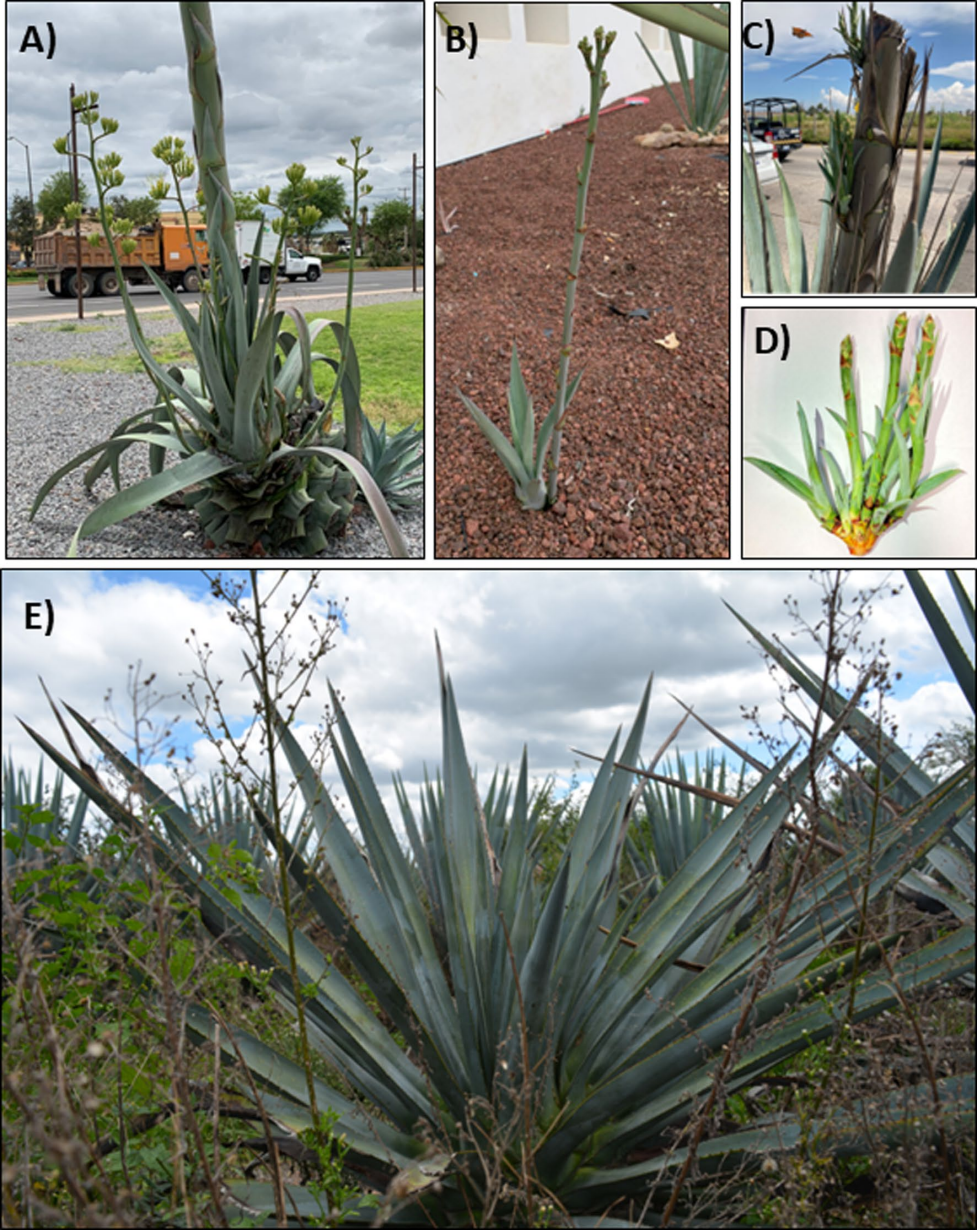
Flowering time in *Agave* species. **A** Agave plant with multiple inflorescences emerging. **B** Inflorescence developed prematurely in a vegetative plant. **C** Inflorescences developing on a mature decapitated inflorescence. **D** Inflorescences developed on bulbils. **E** Agave plant with a permanent vegetative stage, commonly known as “novillo” plants

*Agave* plants flower within a given time frame depending on the species, however plasticity in the timing of flowering can also be observed (Fig. 1). Some plants produce multiple small inflorescences around the main inflorescence (Fig. 1a), and even immature plants have the capacity to produce inflorescences such as offsets that remain attached to the mother plant (Fig. 1b). Inflorescences also form on a mature, decapitated inflorescence (Fig. 1c) and in bulbils formed following flower abortion on the inflorescence. (Fig. 1d). On the other hand, some plants denominated “novillos” undergo the initial “sinking” stage of the flowering process but fail to develop an inflorescence (Fig. 1e). Our hypothesis is that the offsets perceive the signal to undergo the flowering process that has been generated in the mother plant and are stimulated to undergo the flowering process. This suggests that the transition from the vegetative to reproductive stage does not depend on the size or the age of the plant and that juvenile plants have this capacity but lack a positively acting signal. The most probable candidates are activator type FT proteins that are known to be mobile or by carbohydrate signaling.

### Gene structure is conserved between *AtqFTs* and homologues in other species

**Fig. 2:**
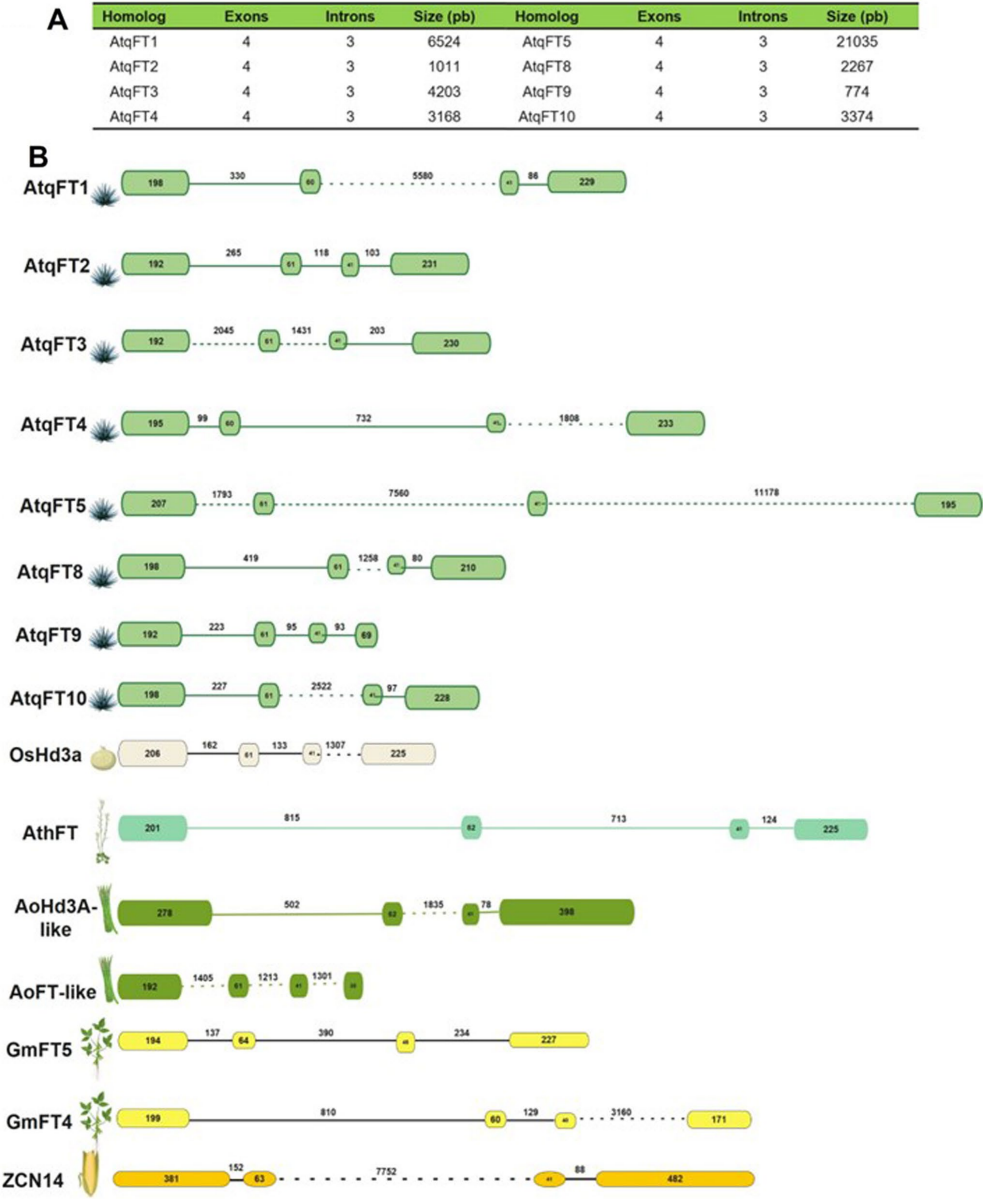
Genomic Structure of *A. tequilana* FTs Homologs. **A** The table summarizes the intron/exon structures of the *AtqFTs*. **B** Schematic representation of the genomic structures. The exons are represented by boxes and introns are represented by lines between the exons. Large introns are represented by dotted lines

The 8 agave FT genomic sequences identified show conserved gene structures typical of the FT gene family that consist of four exons and three introns (Fig. 2). The size of each exon is between 192–207, 60–61, 41, and 195–233 bp, respectively. However, the fourth exon of FT9 is 60 bp. AtqFT5 is the largest gene with a size of 21 kb, due to the presence of three expanded introns.

**Fig. 3:**
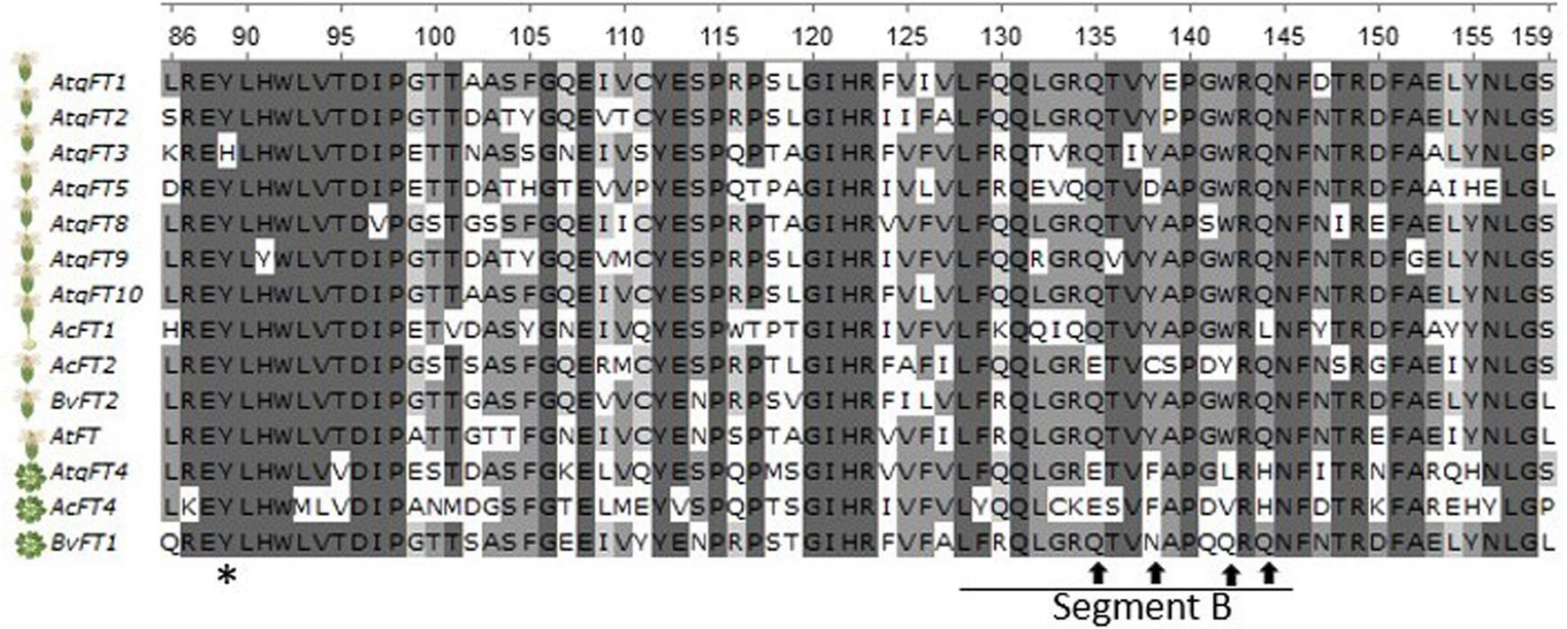
Amino acid sequence alignment of Flowering Locus T. Partial alignment of the Flowering Locus T sequences of *A. tequilana* (AtqFT1 to AtqFT10), *A. thaliana* (AtFT and AtTFL1), *Allium cepa* (AcFT1, AcFT2, and AcFT4), and *Beta Vulgaris* (BvFT1 and BvFT2). The asterisks at the bottom indicate Tyr-89 (Y). The solid black line indicates segment B, the arrows indicate the positions Glu-135 (Q), Phe-138 (F), Leu-142 (L), and His-144 (H), respectively. The activators are represented by flowers while repressors are represented by rosettes

The genomic sequences confirmed the presence of the specific residues conserved among the AtqFT homologues that determine activator/repressor functions. Tyrosine at position 89 (activator function) is present in AtqFT1, AtqFT2, AtqFT4, AtqFT5, AtqFT8, AtqFT9, and AtqFT10, however AtqFT4 has substitutions in critical residues such as Glu-135, Phe-138, Leu-142, and His-144 in the B segment (Fig. 3) consistent with the transcriptome sequences and predictions reported by Ávila de Dios et al. (2019). Repressor FTs from *Beta vulgaris* (BvFT1) and *A. cepa* (AcFT4) also show modifications in the B segment.

### Cis-element analysis of AtqFT promoter regions showed tissue-specific and environmental response motifs

**Fig. 4:**
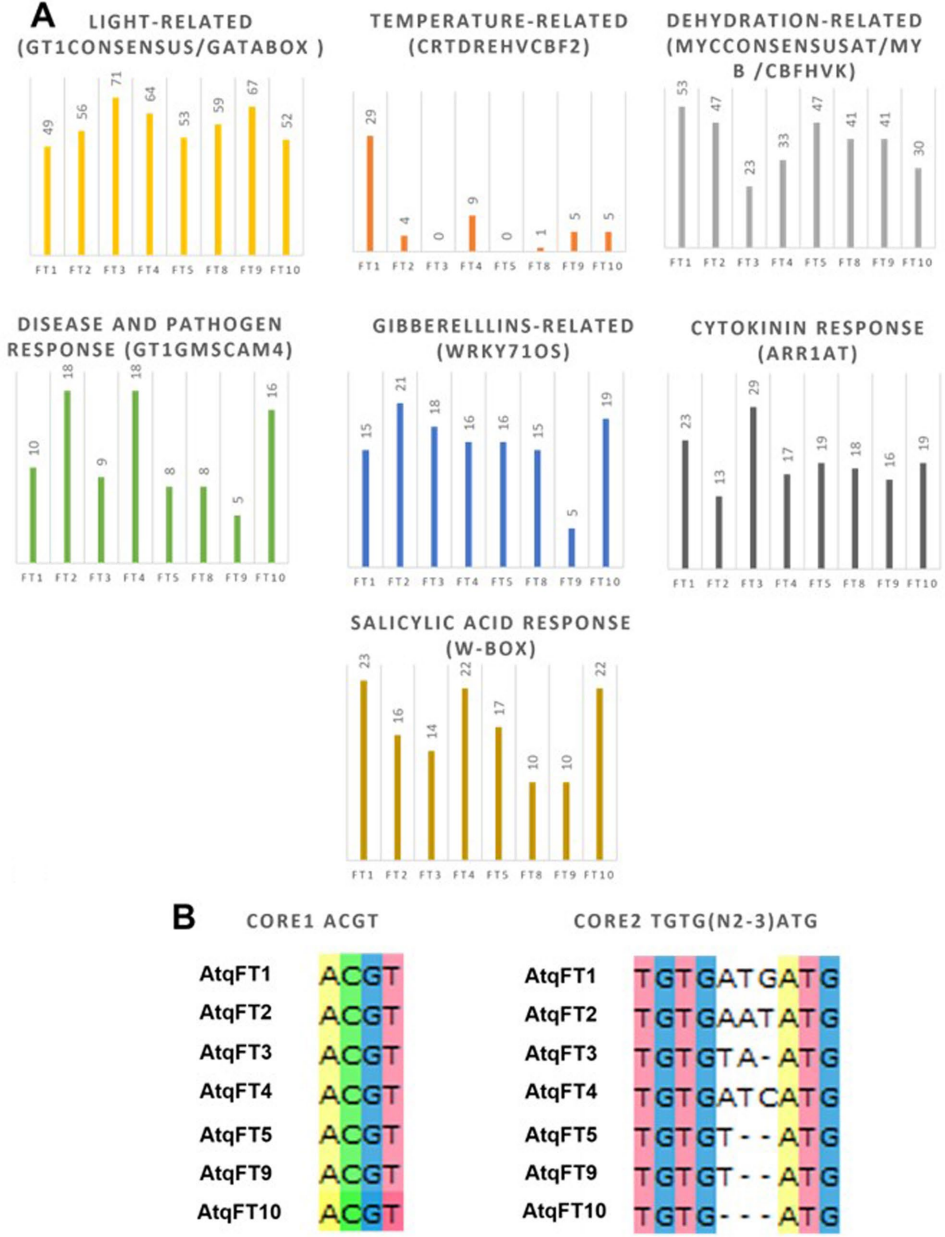

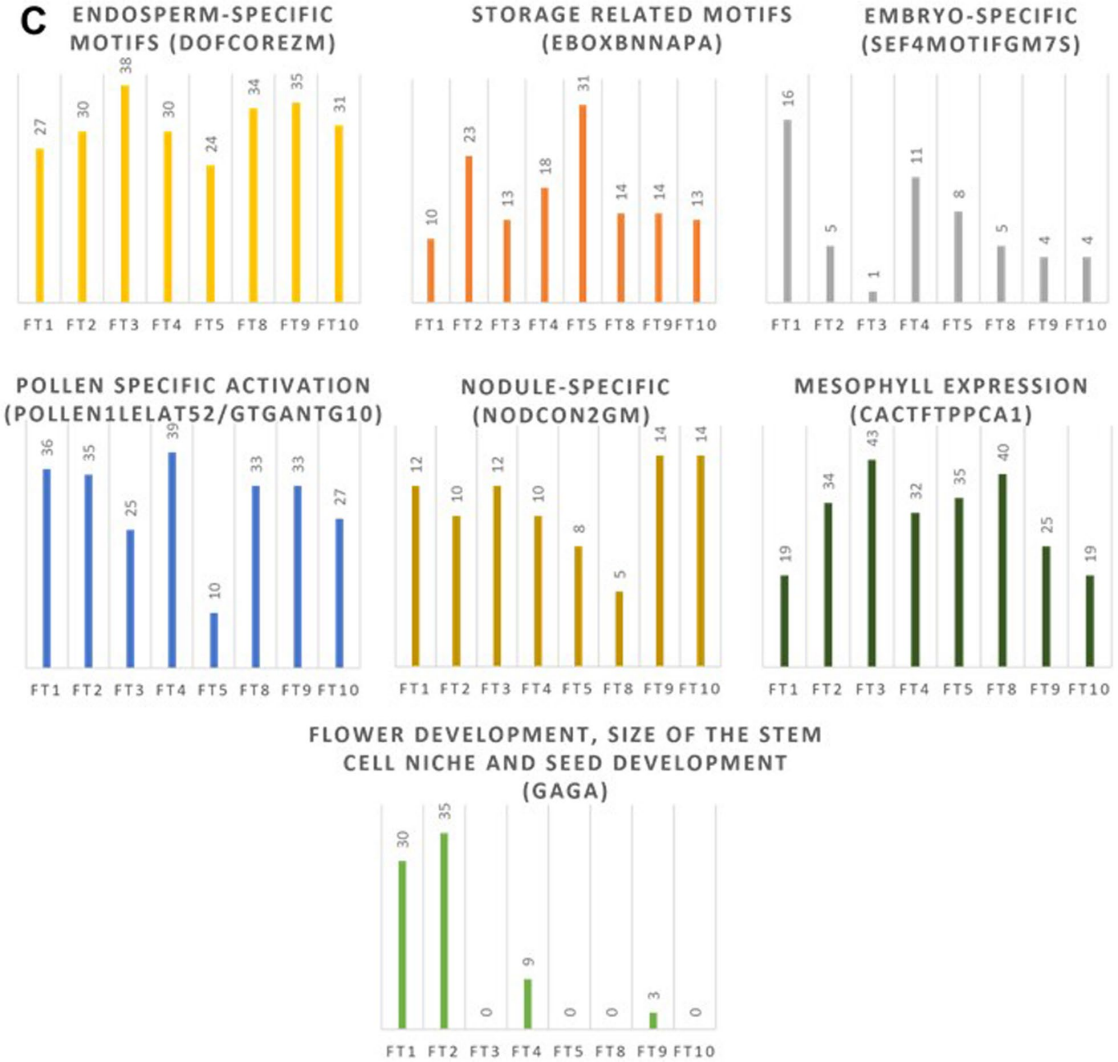
Abundance of transcription factor regulatory motifs identified in AtqFT promoter regions. **A** Cis motifs related with environmental stimulus and hormone signals. **B** Cis motifs related with CONSTANS regulation. C. Cis Motifs Related with development. Conserved motifs and their associated regulatory process are indicated above each graph. AtqFT members are indicated at the foot of each column on the x-axis and the y-axis shows the numbers of motifs identified

Promoter motifs related to environmental stimuli, such as light, temperature, dehydration, and pathogen responses were identified. Motifs related to tissue specific expression such as endosperm-specific, storage, embryo-specific, pollen specific, nodule-specific, mesophyll expression and flower development and to hormone responses (gibberelllins, cytokinins, and salicylic acid) were also identified (Fig. 4).

Light regulatory cis-elements were the most abundant in the AtqFT promoters. GT1CONSENSUS and GATABOX motifs were found in all AtqFTs and two CO-responsive elements CORE1 (ACGT) and CORE2 (TGTG(N2-3) ATG) were also found in all AtqFTs with the exception of AtqFT8. On the other hand, the distal motif CCAAT-box was found in all AtqFT promoters.

Dehydration response motifs were the second most abundant motifs in the promoter regions of AtqFTs, such as MYCCONSENSUSAT, MYB elements, and CBFHVk, although they were found to a lesser extent in AtqFT3. The temperature-related motif CRTDREHVCBF2 was only found in certain homologs such as AtqFT1, AtqFT2, AtqFT4, AtqFT8, AtqFT9, and AtqFT10 although it was most abundant in AtqFT1. The disease and pathogen response GT1GMSCAM4 motif was found in all the AtqFT homologs but to a greater extent in AtqFT2, AtqFT4, and AtqFT10.

Hormone signaling motifs were found in all the *AtqFTs*, including WRKY71OS, ARR1AT, WBOXATNPR1 related to gibberellins, cytokinin, and salicylic acid, respectively.

In addition, DOFCOREZM; EBOXBNNAPA; SEF4MOTIFGM7S; POLLEN1LELAT52; GTGANTG10; and NODCON2GM tissue specific motifs were also found in all AtqFTs. Interestingly, the motif related to flower development, GAGA, was only found in AtqFT1, AtqFT2, and AtqFT4 although in AtqFT4 a smaller number of GAGA motifs were identified in comparison to AtqFT1 and AtqFT2. Results of the analysis of the AtqFT Gene promoter regions are summarized in Online Resource 2.

### Ectopic expression of *AtqFT Genes *modifies flowering time in *A. thaliana*

**Fig. 5:**
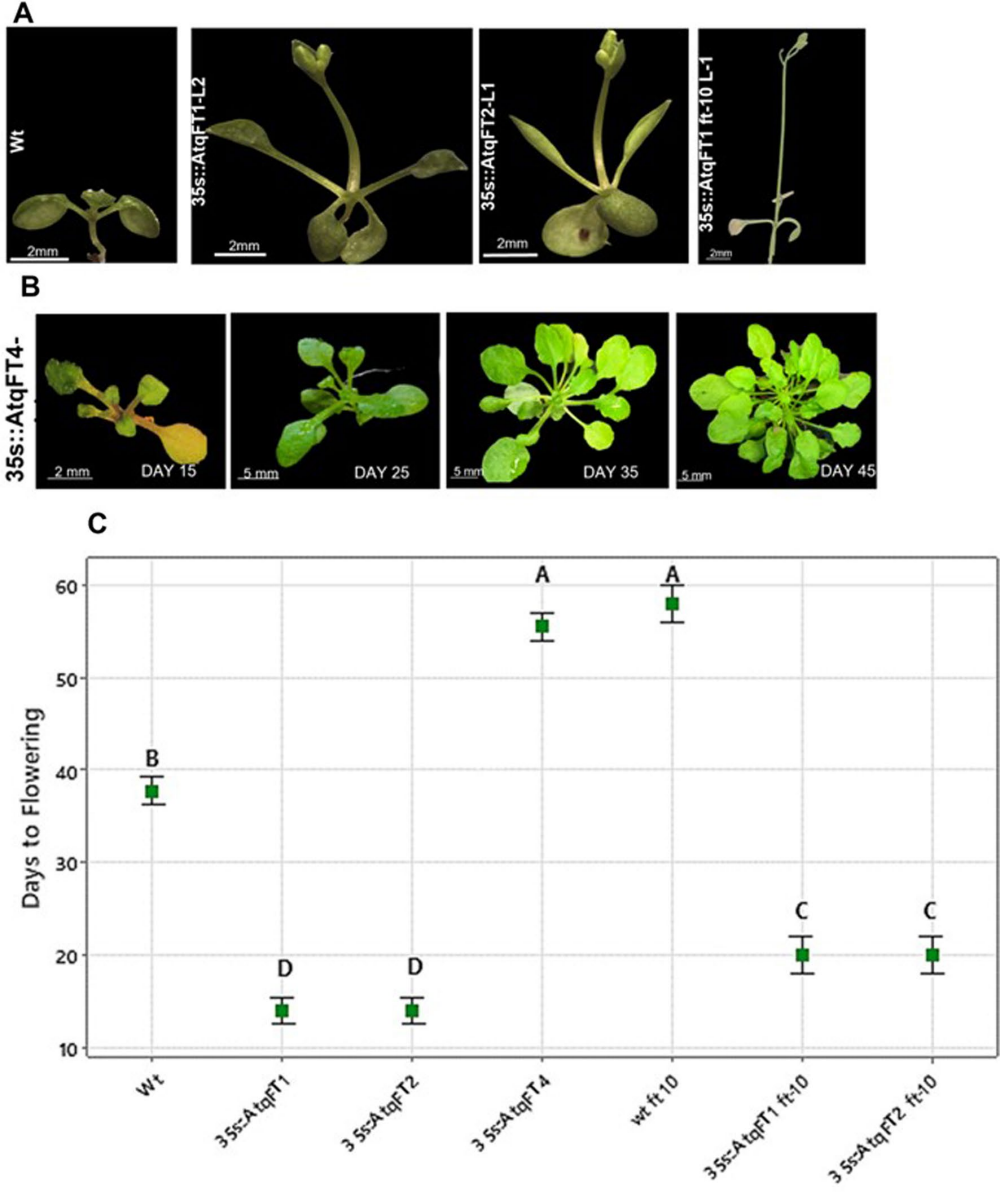
Effect of *AtqFT* overexpression in *Arabidopsis thaliana* col-0 and ft-10. **A** Phenotypes of transgenic lines overexpressing AtqFT1 and AtqFT2 in Wt and *ft-10* mutant plants during long day conditions. **B** Phenotypes of transgenic lines overexpressing AtqFT4 in Wt plants during long day conditions. **C** Comparison of flowering time in transgenic lines, wild type, and* ft-10* mutant plants. Data points represent averages with standard errors for each group. Letters indicate statistically significant differences between the groups (ANOVA, Pb 0.05). Five independent T1 transformants were analyzed for AtqFT1, AtqFT2 and AtqFT4 in the Col-0 background and two T1 independent transformants for AtqFT1, AtqFT2 in the *ft-10* background expression of the *AtqFT4* repressor specifically in phloem tissue

Based on in silico predictions of *A. tequilana* FT gene functions, AtqFT1, AtqFT2, and AtqFT4 were chosen for characterization by expression in the heterologous system of *A. thaliana* ecotype Columbia (Col-0). Nineteen independently obtained 35S::AtqFT T1 lines (7 AtqFT1, 7 AtqFT2, and 5 AtqFT4) were confirmed by PCR. Phenotypes showing altered flowering time were characterized in 5 independent transgenic lines for each gene in the T1 generation by quantifying the days from germination to the appearance of the inflorescence, the number of rosette leaves, and the diameter of the rosette. 35 s::AtqFT1 and 35S::AtqFT2 showed a significantly shorter flowering time with respect to the wild type lines, flowering on average 13.8 days after germination in comparison to the average of 38 days after germination observed for the wild type lines (Fig. 5a, c). 35S::AtqFT1 and 35S::AtqFT2 transgenic lines produced either 2 or 4 small rosette leaves, with in both cases an average diameter of 1.3 cm (Table 1). The transgenic lines showed severe phenotypes and died in less than 25 days. No viable seeds could be obtained from these lines and further generations could not be produced. 35S::AtqFT4 T1 transgenic lines also showed strong phenotypes and produced few viable seeds. The 35S::AtqFT4 T1 lines flowered on average, 55.2 days after germination demonstrating a significantly delayed flowering time in comparison with wild type plants (Fig. 5b, Table 1). The phenotype of 35S::AtqFT4 plants was similar to the *ft-10* mutant line which flowered on average after 58 days. These results suggest that the in silico predictions for the function of 35S::AtqFT1 and 35S::AtqFT2 as promoters of flowering and that of 35S::AtqFT4 as a repressor of flowering are accurate.

**Table 1:** Effects of overexpression of *AtqFT genes* in *Arabidopsis thaliana* col-0 and ft-10

Genotype	Days to flowering	Number of leaves	Diameter cm
35s::FT1			
FT1-T1-15	15	4	1.1
FT1-T1-16	15	4	1.4
FT1-T1-17	13	2	1
FT1-T1-18	13	2	1.5
FT1-T1-19	13	2	1.5
35s::FT2			
FT2-T1-18	15	4	1.4
FT2-T1-19	13	2	1.6
FT2-T1-20	15	4	1.1
FT2-T1-21	13	2	1
FT2-T1-22	13	2	1.4
35s::FT4			
FT4-T1-7	56	30	3.8
FT4-T1-8	55	31	3.7
FT4-T1-9	55	31	3.5
FT4-T1-10	56	30	3.8
FT4-T1-11	54	32	4.5
Wt			
Wt	35	19	4
Wt	39	14	4
Wt	38	14	3
Wt	39	14	4
Wt	39	15	3
35s::AtqFT1 ft-10			
L1	20	4	1.1
L2	20	4	1.3
35s::AtqFT2 ft-10			
L1	20	4	1.4
L2	20	4	1.8
ft-10			
ft 10-1	60	38	6.5
ft 10-2	56	36	7.2

Five independent lines with ectopic expression of each AtqFT gene: AtqFT1, AtqFT2, and AtqFT4, five wild type, non-transformed lines and 2 independent transgenic lines for *AtqFT1, AtqFT2 in the ft-10* mutant background were analyzed

In order to confirm the function of the AtqFTs, the *A. thaliana* ft-10 mutant line was also transformed with the 35S::AtqFT1 and 35S::AtqFT2 and 35S::AtqFT4 constructs. Two independent transgenic lines were obtained for AtqFT1 and AtqFT2. For both constructs a significant reduction (even lower than the wild type) in flowering time in relation to the *ft-10* mutant plants was observed although this was longer than the flowering time of the wild type lines transformed with the 35S::AtqFT1 and 35S::AtqFT2 (Table 1, Fig. 5c). These results indicate that the 35S::AtqFT1 and 35S::AtqFT2 constructs expressed ectopically in *A. thaliana* can rescue the late flowering phenotype of the *ft-10* mutant. 35S::AtqFT4 lines could not be obtained using the *ft-10* background. Since 35S::AtqFT4 has a repressor function and the *ft-10* mutant also leads to delayed flowering, it is possible that the combination of both repressor effects lead to a non-viable phenotype. Together, these findings confirm that AtqFT1 and AtqFT2 are promoters of flowering whereas AtqFT4 acts as a repressor of flowering and also indicate that the AtqFT genes can interact with the endogenous components of the *A. thaliana* FT regulatory system to either promote or inhibit flowering, even though *A. thaliana* does not employ the strategy of antagonistic FT genes shown to be present in *B. vulgaris*, *A. cepa* and now *A. tequilana*.

### Differential expression of *AtqFTs* in vascular tissue of *A. tequilana* Leaves

Reports in *A. thaliana* have shown that AtFT is specifically expressed in the companion cells of phloem tissue of leaves (Corbesier et al. 2007), and that the FT protein is then transported through the phloem to the shoot apical meristem where it interacts with other proteins to induce the initiation of flowering.

**Fig. 6:**
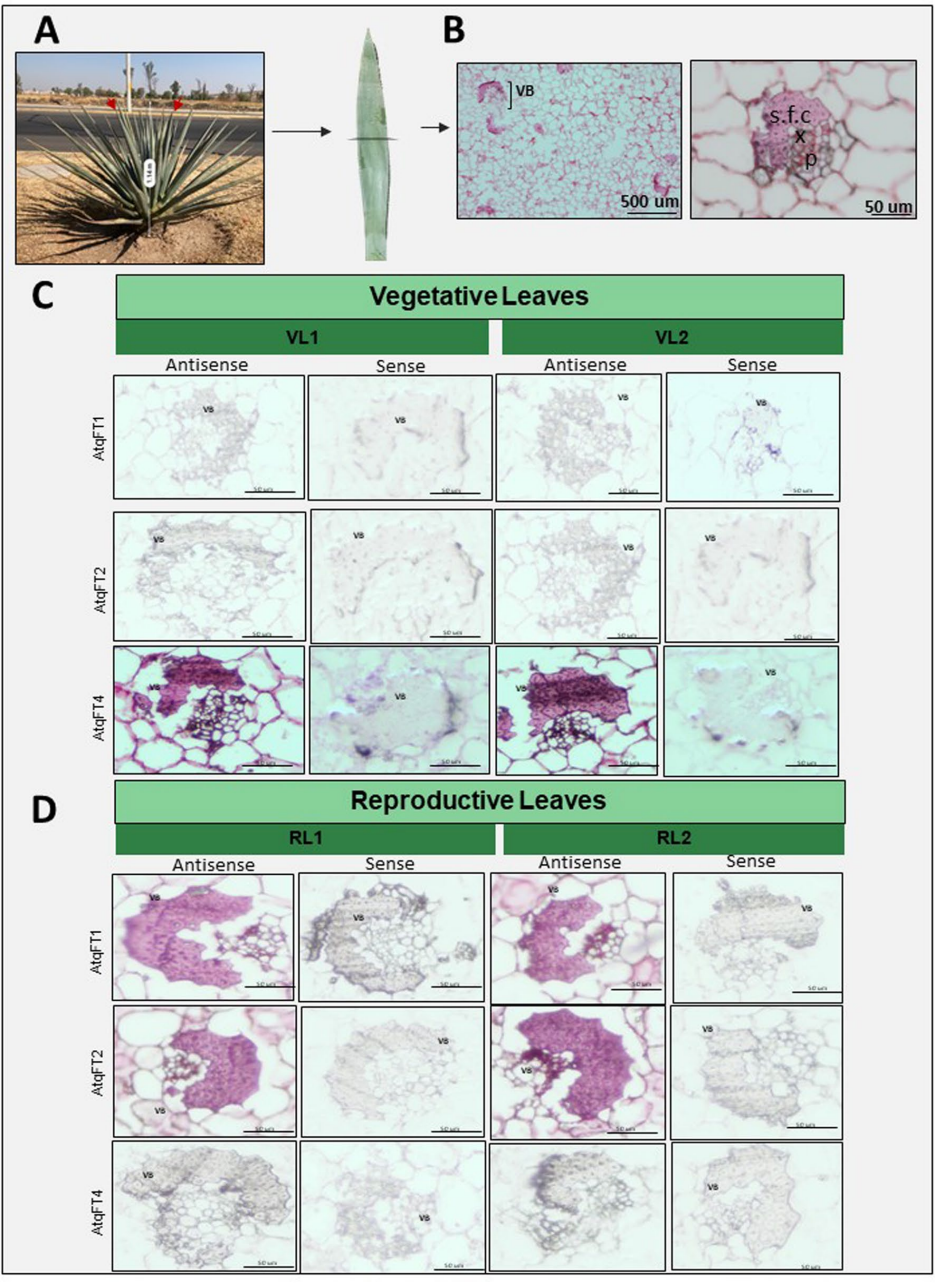
In situ hybridization analysis. **A** Samples were collected from the mid-section of the third layer of fully opened leaves below the SAM. Arrows indicate the leaves collected. **B** Transverse section of *Agave tequilana* leaves stained with periodic acid Schiff (PAS) reaction to identify vascular bundles (VB); Sclerenchymatous fiber cap (s.f.c); xylem (x); and phloem (p). **C** Transversal section of vegetative leaves hybridized with AtqFT1, AtqFT2, and AtqFT4-specific antisense and sense probes. **D** Transversal section of bolting stage leaves were hybridized with AtqFT1, AtqFT2, and AtqFT4-specific antisense and sense probes. Scale bars on in situ hybridization analysis are 50 um

In order to determine whether AtqFT1, AtqFT2, and AtqFT4 are also specifically expressed in phloem tissue, in situ hybridization analysis was carried out in transversal sections of leaves and meristem tissues of *A. tequilana* plants at the vegetative and bolting stages of development (Fig. 6). Sense riboprobes were used as control in order to determine the specificity of the hybridizations. The in situ hybridization analysis shows that AtqFT1, AtqFT2, and AtqFT4 are all expressed in the sclerenchymous fiber cap and phloem tissues in leaves whereas none of the genes are expressed in SAM tissue at either of the developmental stages tested. No signal was detected in other cell types. The AtqFT4 signal was stronger in vegetative leaves, suggesting the expression of AtqFT4 specifically in phloem and sclerenchymous fiber cap during the vegetative stage. AtqFT1 and AtqFT2 showed no signal in vegetative leaves. However, hybridization of AtqFT1 and AtqFT2 probes can be observed in phloem and sclerenchymous fiber of leaves at the bolting stage whereas AtqFT4 is not observed at this stage. This suggests that the expression of AtqFT4 decreases in the bolting stage while the expression of AtqFT1 and Atq1FT2 increase. The in situ hybridization results also support the roles of AtqFT1, AtqFT2, and AtqFT4 as promoters and repressors of flowering, respectively, and the hypothesis that both activator and repressor type FTs can be transported to the SAM in order to carry out their function as evidenced by the expression of the AtqFT4 repressor specifically in phloem tissue.

### Identification of members of the SPL and AP2 gene families of *A. tequilana*

*Agave* species are monocarpic perennials with long life cycles and for many years ignore the normal environmental cues such as photoperiod and temperature which lead to flowering. Therefore it is probable that the age-related and/or endogenous flowering pathways (that involve regulation by miRNAs and sugar signaling), play a significant role in the vegetative to reproductive transition in these species. Based on data from *A. thaliana* and other plant species it was expected that members of the SPL and AP2 gene families would harbor target sequences for miR156 and miR172, respectively. Therefore *A. tequilana* transcriptome data-bases were analyzed to identify members of the SPL and AP2 gene families in *A. tequilana* and to then search for the miRNA target sequences.

### AtqSPL family

Analysis of *A. tequilana* transcriptome data (Ávila de Dios et al. 2019) identified 13 putative full-length transcripts encoding SPL proteins. The deduced amino acid sequences of these proteins were aligned with SPL protein sequences from different monocotyledonous and dicotyledonous species and the dendrogram presented in Online Resource 4 was constructed. *A. tequilana* SPL genes were assigned names based on their association with previously characterized SPL genes from *A. thaliana* within the dendrogram. Based on previous reports in *A. thaliana*, AtqSPL2, AtqSPL8, AtqSPL9, AtqSPL12 and AtqSPL13 were predicted to harbor target sites for miR156 and a more detailed analysis was carried out to identify conserved motifs within the protein/nucleotide sequences. As can be observed in Online Resource 5, the motifs found in the *A. tequilana* sequences are consistent with those found in other species such as *O. sativa*, *A. thaliana* and *M. domestica* (Jiang et al. 2021). A 6th SPL gene AtqSPL6 harboring a miR156 target sequence was also identified however since this gene does not contain motifs 1 and 2 it was not considered for expression analysis.

### AtqAP2 family

A search of the *A. tequilana* transcriptome database led to the identification of 305 sequences with homology to the AP2-like family. However, the majority (69%) encoded RAV or ERF type proteins harboring a single AP2 domain that do not participate in the vegetative to reproductive transition. The remaining (31%) sequences corresponded to sequences encoding 2 AP2 domains (AP2-R1 and AP2-R2) and 3 *A. tequilana* AP2 candidate cDNAs were identified 2 of which correspond to full-length cDNA sequences and the 3rd although it covers the 2 AP2 domains is a truncated sequence. Alignment of the deduced amino acid sequences and comparison with AP2 sequences from other plant species led to the construction of the dendrogram in Online Resource 6. As can be observed 2 of the deduced proteins group within a clade containing AP2 proteins from rice (*Oryza sativa*) and banana (*Musa acuminata*) and the third candidate groups with the related aintegumenta (ANT) protein from *A.thaliana*, *M. acuminata* and *Asparagus officinalis*.

Sugars are also known to regulate miR156 and play a role in the age-related pathway that determines the vegetative to reproductive transition (Yang et al. 2013). Previously published data (Ávila de Dios et al. 2019) for members of *A. tequilana* Plant Glycoside Hydrolase Family 32 (PGHF32) which contains vacuolar and cell wall invertases and enzymes involved in fructan metabolism, was accessed in order to identify putative miRNA target sequences in these gene families.

### Identification of miRNA targets in AP2, SPL and PGH32 gene families

Analysis of *A. tequilana* transcriptome data identified 46 conserved miRNA families (Online Resource 7). The 3 *A. tequilana* gene families encoding SPL, AP2 and PGHF32 were therefore analyzed to identify the presence of putative target sequences for each of these miRNAs. The results for miRNAs identified in each gene family are summarized in Online Resources 8 and 9. As can be observed for the AtqSPL family, target sequences for 6 different miRNAs were identified in 3 or more genes including as expected miR156, which is present in 6 of the 12 AtqSPL genes. Target sequences for 13 other miRNAs were identified in only 1 or 2 AtqSPL genes including miR172, which normally has an antagonistic function in relation to miR156, however targets for both miR156 and miR172 were identified in AtqSPL13. Although the data for the AtqAP2 family are not strong since only 2 members were identified and 1 is probably incomplete, as expected a target sequence for miR172 was identified for AtqAP2 and no targets for miR156 were observed for either of the AtqAP2 genes.

In the case of the PGHF32 gene family, 6 different miRNA targets were also identified in at least 3 genes of the family and a further thirteen were identified in either 1 or 2 genes. Interestingly targets for miR156 were identified for 7 different *A. tequilana* PGHF32 genes including the 3 Atq1-SST genes and vacuolar and cell wall invertase genes. miR156 targets were not identified in any of the AtqFEH genes and miR172 targets were also absent from the PGHF32 genes. Another interesting result was the presence of miRNA targets for miR396 in exohydrolase and vacuolar invertase genes but not in fructan synthesis genes or in cell wall invertase genes (Online Resource 9).

Comparing the 3 gene families, targets for miRNAs 166 and 159 are shared across all 3 families. The SPL and PGHF32 families share 6 miRNA targets including those for miR156 and miR396. In contrast only a single miR2118 target is shared between PGHF32 and AP2 and only miR172 and miR408 targets are shared between SPL and AP2 families.

In order to determine whether the miRNA targets identified for *A. tequilana* PGHF32 genes were also present in PGHF32 members from other plant species, PGHF32 genes from *A. thaliana*, sugar beet (*Beta vulgaris*), asparagus (*A. officinalis*) and onion (*Allium cepa*) were examined. Based on this analysis miR165 and miR164 were only found in PGHF32 genes from *A. tequilana.* In contrast miR172, miR482 and miR8558 were found in at least 3 other species but not *A. tequilana*. miR396 was identified in all species with the exception of onion, miR159 in all species with the exception of asparagus and miR166 in all species.

### In silico expression patterns of miRNAs 156, 172 and 164 and their target genes

**Fig. 7:**
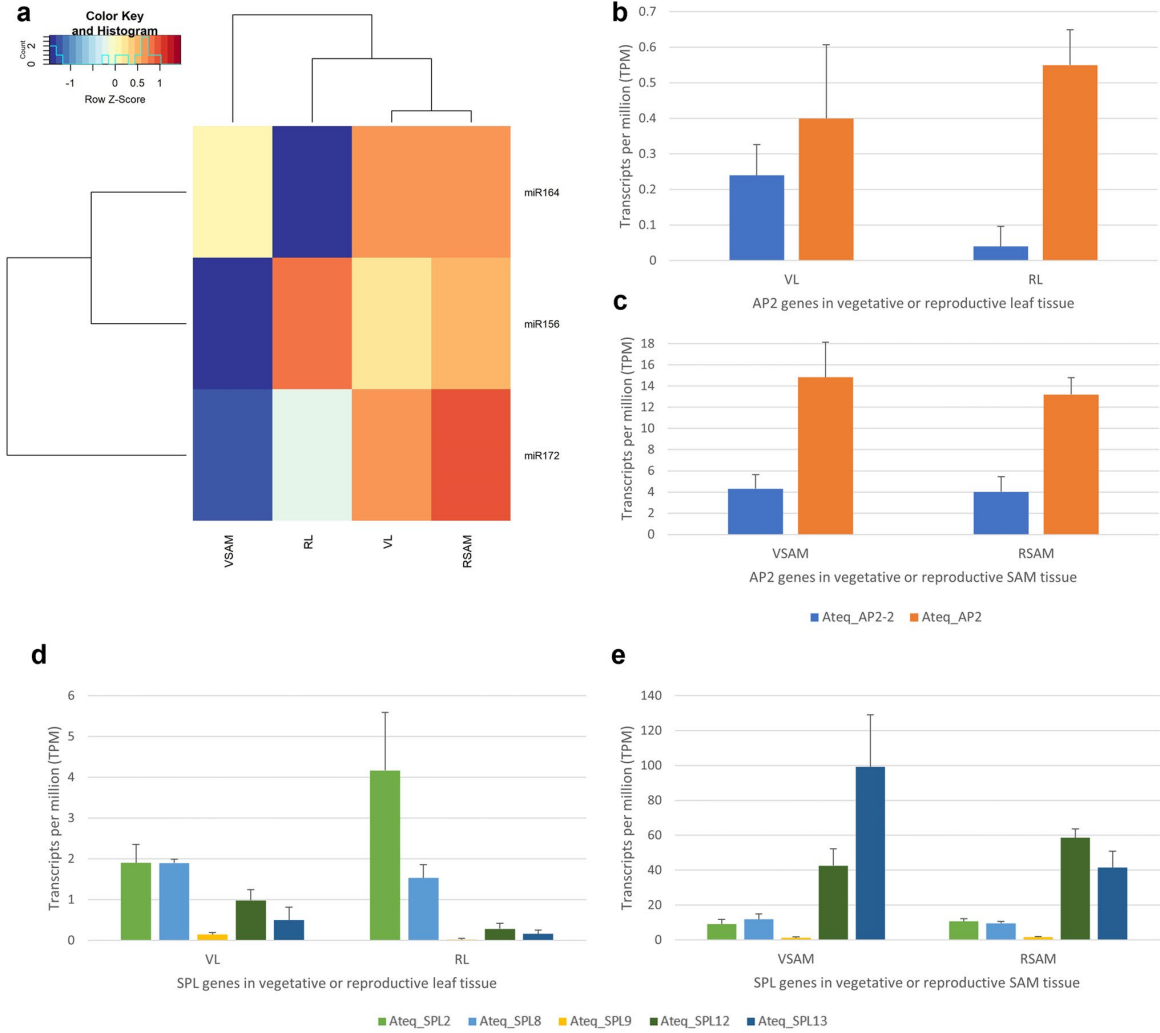
In silico expression patterns of miRNAs and their target genes. **A** Heat diagram of in silico expression patterns of miR156, miR164 and miR172. Red indicates higher number of transcripts, whereas blue indicates lower number of transcripts. **B**, **C** Expression patterns of AtqAP2 genes in vegetative and reproductive leaf and SAM tissue. **D**, **E** Expression patterns of AtqSPL genes in vegetative and reproductive leaf and SAM

miRNAs 156, 172 and 164 were selected in order to determine whether their expression patterns corresponded to the genes containing their target sequences based on in silico data for vegetative leaf and SAM tissue (VL, VSAM, respectively) and leaf and SAM tissue from the first detectable stage following the vegetative to reproductive transition (RL, RSAM, respectively) (Fig. 7a). Both AtqAP2 targeted by miR172 and AtqAP2-2 with no target sequence, are expressed at very low levels in VL and RL tissue. In SAM tissue AtqAP2 shows higher expression in VSAM tissue in comparison to RSAM tissue whereas AtqAP2-2 shows no difference in expression. The expression pattern of miR172 coincides with that of AtqAP2 in SAM tissue showing low expression in VSAM and high expression in RSAM (Fig. 7b, c).

The AtqSPL family genes targeted by miR156 show different patterns of expression. AtqSPL2 and 8 are expressed in both VL and RL tissue although AtqSPL2 shows a slightly higher level of expression in RL. AtqSPL 9, 12 and 13 are expressed at low levels in VL tissue and this expression is even lower in RL tissue. In contrast, AtqSPL2, 8 and 9 are expressed at low levels in both VSAM and RSAM whereas AtqSLP12 and 13 are expressed strongly in VSAM tissue and while AtqSLP12 shows a higher level of expression in RSAM tissue, AtqSPL13 shows a drop in expression in RSAM. The AtqSPL13, 12 and 9 gene expression patterns correspond to the miR156 pattern in leaf tissue being highest in RL tissue and lower in VL tissue and AtqSPL13 also corresponds to the miR156 pattern in SAM tissue: low in VSAM and higher in RSAM. AtqSPL12 however although expressed in VSAM shows higher expression in RSAM (Fig. 7d, e).

**Fig. 8:**
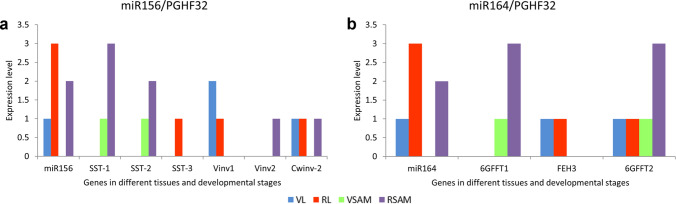
Relation of expression patterns in microRNAs and PGHF32 genes. Expression of **A** miR156 and **B** miR164 genes compared to AtqPGHF32 genes in vegetative and reproductive leaf and SAM tissue. Data modified from Ávila de Dios et al. 2019

The expression data for PGHF32 has been published previously and was used to compare the expression patterns with those of the selected miRNAs. The original heatmap data was converted to a scale of 0–3 to compare with the miRNA expression data and obtain the data presented in Fig. 8. miR156 is expressed in VL, RL and RSAM tissue as can be observed in Fig. 8a and AtqSST-1 and 2 and AtqVinv1, PGHF32 genes which harbor miR156 targets are not expressed in VL or RL corresponding to this pattern. These genes are expressed however in VSAM where miR156 is not expressed and are strongly expressed in RSAM. AtqVinv1 and 2, Atq1SST-3 and AtqCwinv-2 although also harboring miR156 target sites do not show corresponding expression patterns. miR164 shows the same pattern of expression as miR156 in VL, RL and RSAM tissue and the expression of Atq6GFFT1 and 2 and AtqVinv1 which harbor target sequences for miR164 also coincides with this pattern showing expression in VSAM tissue (Fig. 8b). In a similar fashion to AtqSST-1 and 2 and AtqVinv1 in relation to MiR156, Atq6GFFT1 and 2 and Atqinv1 are also expressed in RSAM tissue. AtqFEH3 shows the same expression pattern as miR164 in leaf but not SAM tissue and Atq6GFFT2 although expressed in both VSAM and RSAM tissue is also expressed in VL and RL tissue. It may be the case that although miR156 and miR164 are expressed in RSAM and VSAM, their level of expression may be too low to disrupt the expression of the genes containing their targets in this tissue.

## Discussion

### Genome analysis of the *A. tequilana* FT/TFL family

The homologs previously reported by Ávila de Dios et al. (2019), namely AtqFT1, AtqFT2, AtqFT3, AtqFT4, AtqFT5, AtqFT8, AtqFT9, and AtqFT10 were found in the *A. tequilana* draft genome, and the putative exon/intron structures were determined. The sequences identified showed conserved gene structures typical of the FT gene family, consisting of four exons and three introns. Genome coordinates for the AtqFT6 and AtqFT11 transcripts described by Ávila de Dios et al. (2019) were the same for AtqFT8 and AtqFT4, respectively. This suggests that AtqFT6 and AtqFT11 are not distinct FT loci but alleles of AtqFT8 and AtqFT4, respectively. On the other hand, Genome coordinates for the AtqFT7 pseudogene were not found suggesting that the original annotation of this gene may have been erroneous, perhaps due to inconsistencies in the assembly process.

Although some differences in certain amino acids between the transcriptomic and genomic sequences were found, the residues that determine the activator/repressor functions within the FT homologs are consistent with the predictions reported by Ávila de Dios et al. (2019). Therefore, AtqFT1, AtqFT2, AtqFT4, AtqFT5, AtqFT8, AtqFT9, and AtqFT10 possess residues present in activator homologs whereas AtqFT4 has substitutions in the B segment suggesting a role as a repressor.

### Detailed analysis of the FT B segment

The biennial crops Onion (*Allium cepa*) and sugar beet (*Beta vulgaris*) in common with *A. tequilana* harbor both promoter and repressor type FT genes. For example, following germination AcFT4 inhibits bulbing during spring until a long day photoperiod is reached when AcFT4 expression declines and AcFT2 expression increases leading to bulb formation. The plant is later subjected to cold winter temperatures leading to the expression of AcFT1 that induces flowering in the second spring period (Lee et al. 2013). Similarly in sugar beet, BvFT1 represses flowering whereas BvFT2 induces flowering (Pin et al. 2010). *A. cepa* and *A. tequilana* are both members of the Asparagaceae family and the repressor type FTs of these species: AtqFT4 and AcFT4 share amino acid changes in the B segment which define the activator or repressor function. The repressor from B. vulgaris BvFT1 shows changes at the same amino acids as AtqFT4 and AcFT4 although the type of amino acid is not conserved. Interestingly, AcFT2 that promotes bulbing but not flowering in onion also shows modifications consistent with repressor function in the B segment. These observations suggest that a similar mechanism involving activator and repressor FTs has evolved separately in taxonomically distant plant families (Asparagaceae and Amaranthaceae) to control flowering time and/or bulbing in a biannual or perennial monocarpic mode.

These results contrast with reports from *Bamboo* species many of which, in common with *Agave* species are monocarpic perennials. The FT regulatory pattern in bamboo is similar to that described in *A. thaliana* and does not involve the antagonistic expression of activator and repressor FTs described above for agave, onion and sugar beet. For example, in *Phyllostachys heterocycla* five members of the FT/TFL family were identified. PhFT4 promotes flowering in *A. thaliana*, whereas PhFT1, PhFT2 and PhFT3 suppress flowering. However in this case PhFT4 was the only FT gene identified, whereas PhFT1, PhFT2, and PhFT3 are TFL1 type genes (Yang et al. 2019). In *Phyllostachys violascens*, two FT-like genes, PvFT1 and PvFT2, were identified and PvFT1 was shown to be a promoter of flowering whereas PvFT2 is thought to be involved in other developmental processes (Guo et al. 2016).

These observations in agave, Arabidopsis, bamboo, onion and sugar beet, confirm that a variety of strategies for the regulation of flowering by FT family genes have evolved independently and are not directly related to the taxonomy of the species, the monocarpic or polycarpic mode of flowering or annual and perennial lifecycles.

### Analysis of promoter regions of *A. tequilana* FT genes

Overall, no regulatory motifs were exclusive to either promoter or repressor type FT genes however GAGA motifs related to regulation of flowering were only found in AtqFT1, AtqFT2, and AtqFT4. These findings are consistent with the proposed roles of AtqFT1, AtqFT2, and AtqFT4 as promoters and repressors of the vegetative to reproductive transition. However, within the activator genes, numbers of motifs varied widely and could indicate modulation of specific FT genes under different environmental conditions or in response to hormones. A striking example is the presence of the temperature-related motif CRTDREHVCBF2 in significantly more copies in AtqFT1 in comparison to the other *A. tequilana* FT genes. The repressor gene AtqFT4 also has an increased number of copies of this motif in relation to the other FT activator genes although almost 3X lower than AtqFT1. These observations may indicate that temperature could be an important factor in the determination of the onset of the vegetative to reproductive transition in *A. tequilana* as observed in *A. thaliana*, where depending on temperature, PHYTOCHROME INTERACTING FACTOR 4 (PIF4) promotes the expression of FT by binding directly to the promoter region (Cho et al. 2017).

Based simply on the number of motifs associated with different environmental or hormonal effects, it is evident that light is the most important factor affecting the regulation of all *A. tequilana* FT genes. This is similar in *A. thaliana*, where all regulatory elements required to mediate the expression of FT in response to photoperiod were found in the FT promoter, including sequences required for FT activation by CONSTANS (CO) (Cao et al. 2014) (Adrian et al. 2010). GT1CONSENSUS (Kaur et al. 2017) and GATABOX (Gilmartin et al. 1990), light response motifs, which were found in all AtqFTs. Moreover, the cis-element CO-responsive elements CORE1 (ACGT) and CORE2 (TGTG(N2-3) ATG) were also identified in *A. tequilana* FTs. CO encodes a transcription factor that positively regulates FT expression in the photoperiod pathway (Suárez-López et al. 2001) and CORE1 (ACGT) and CORE2 (TGTG(N2-3) ATG) are required to recruit CONSTANS (CO) to the FLOWERING LOCUS T promoter in *A. thaliana* (Tiwari et al. 2010). CORE1 (ACGT) was found in all the AtqFT homologs except in AtqFT8, whereas CORE2 (TGTG(N2-3) ATG) was only found as a complete sequence in the AtqFT1, AtqFT2, AtqFT3 and AtqFT4 promoters. According to Adrian et al. (2010), mutation in CORE2 could eliminate both CO binding and synthetic promoter activation. Thus, it is possible that AtqFT5, AtqFT9, and AtqFT10 would not be activated by CONSTANS due the presence of an incomplete CORE2.

These light-related motifs were also found in the promoter of an FT homolog, GmFTL2 in soybean. However, this homolog seems not to be related to flowering time (Liu et al. 2017).

Dehydration-related motifs are also abundant in all *A. tequilana* FT genes. Although response to dehydration is not considered as a separate floral transition pathway, a previous report showed that drought causes an early arrest of floral development, leads to sterility, and causes differential expression of Flowering Locus T in *A. thaliana* (Su et al. 2013). The presence of significant levels of dehydration-related motifs in *A. tequilana* FT promoters is perhaps surprising since these plants are adapted for growth in arid climates and live for 5–7 years under water stressed conditions without undergoing the vegetative to reproductive transition.

Hormone signaling motifs such as gibberellin, cytokinin, and salicylic acid-related motifs were found in all the *AtqFTs*. The main gibberellin response motif found in AtqFT homologs is WRKY71OS, binding site of WRKY71, a transcriptional repressor of the gibberellin-signaling pathway in rice. ARR1AT, a binding site of cytokinin regulated transcription factor ARR1 and WBOXATNPR1, (W-box) a salicylic acid inducible binding of WRKY were also found (Tiwari et al. 2015).

Tissue specific motifs such as DOFCOREZM; EBOXBNNAPA; SEF4MOTIFGM7S; POLLEN1LELAT52 and GTGANTG10; NODCON2GM; CACTFTPPCA1 related with endosperm-specific Dof proteins, storage proteins, embryo-specific expression, pollen specific activation, nodule expression, and mesophyll expression, respectively (Li et al. 2014) (Tiwari et al. 2015) (Stougaard et al. 1990) were also identified in all the *AtqFTs*. Endosperm-specific, root-specific, and meristem-specific motifs were also reported in the promoter region of soybean *GmFTL2* (Liu et al. 2017). Tissue-specific motifs have also been reported in other members of the PEBP family, such as HbMFT1, a homolog of MFT from Rubber Tree (*Hevea brasiliensis*) (Bi et al. 2016).

Fewer GAGA motifs were found in AtFT4 in comparison to AtqFT1 and AtqFT2. This motif is associated with VERNALIZATION2 (VRN2) and flower development in *A. thaliana* (Hecker et al. 2015). Furthermore, GAGA motifs are recognized by OsGBP1, a gene in rice (*Oryza sativa*) that functions as a transcription factor, whose overexpression showed reduced grain length and seedling growth and led to delayed flowering time (Gong et al. 2018) supporting the hypothesis that AtqFT1, AtqFT2, and AtqFT4 play important roles in regulation of flowering time in *A. tequilana*.

### Functional characterization of *A. tequilana* FT genes

Ectopic expression of AtqFT1 and AtqFT2 in the heterologous system of *A. thaliana* significantly reduced flowering time, confirming the prediction that they are activator type FTs. In contrast, AtqFT4 delayed flowering time in *A. thaliana*, confirming that this gene encodes a repressor type FT protein. These results are also supported by the analysis of the transformed *A. thaliana* *ft-10* mutant line where expression of AtqFT1 and AtqFT2 led to flowering times lower than in the wild type and slightly higher than in the AtqFT1 and AtqFT2 ectopic lines. The AtqFT4 expressing lines produced phenotypes similar to the *ft-10* mutant line suggesting that AtqFT4 interferes with the activity of the *A. thaliana* FT gene (AtFT) that normally promotes flowering. This is an interesting result since *A. thaliana* does not harbor repressor type FT genes other than the TFL type. Since the B segment in AtqFT4 shows modifications in specific amino acids defining the repressor function, it is possible that AtqFT4 prevents the correct interactions between AtFT and the FD and 14–3–3 proteins in the SAM that lead to initiation of the flowering process.

In contrast AtqFT1 and AtqFT2 induce early flowering in the *ft-10* mutant line suggesting that these proteins must also interact with the regulatory proteins in the SAM where their strong ectopic expression leads to the transition to flowering in a shorter time than in wild type plants.

Based on in silico data (Ávila De Dios et al. 2019), AtqFT4 is strongly expressed in vegetative leaves and its expression decreases during the bolting and flowering stages, whereas AtqFT1 and AtqFT2 show an inverse pattern of expression. The in situ hybridization analysis supports the specific expression of the AtqFT1, AtqFT2, and AtqFT4 genes in the sclerenchyma and phloem tissue in leaves but no evidence for expression of AtqFT1, AtqFT2, and AtqFT4 in SAM tissue was uncovered. These observations support the hypothesis that both repressor and activator type FT proteins are synthesized in the phloem of leaf tissue and then transported to the SAM where they are functional. AtqFT4 is strongly expressed in vegetative leaf tissue consistent with a role in repression of flowering during the vegetative and juvenile stages of development whereas AtqFT1 and 2 are more highly expressed in leaf tissue of plants which have undergone the vegetative to reproductive transition, consistent with a role in the activation of flowering. These results are similar to reports of expression of FT activator/repressor homologs in other species such as *Beta vulgaris*, (Pin et al. 2010) and *Pisum sativum* (Hecht et al. 2011).

### Identification of *A. tequilana* SPL and AP2 gene families and putative target sequences for miRNAS involved in regulation of flowering.

To determine how regulatory elements previously described in other plant species could be associated with the FT regulatory model in particular through the ageing pathway, the putative involvement of the AP2/miR172 and SPL/miR156 modules and a possible link between miRNA regulation and carbohydrate metabolism was explored. Transcriptome analysis successfully identified 13 full-length AtqSPL transcripts, 5 of which were predicted to contain the expected target sequence for miR156 (AtqSPL2, AtqSPL8, AtqSPL9, AtqSPL12 and AtqSPL13) based on results obtained in other plant species. However a sixth AtqSPL transcript (AtqSPL6) containing a miR156 target was also identified but was not included in subsequent analysis since the structure of this gene differed from SPL genes known to be associated with the flowering process. Perhaps surprisingly, miR172 (normally antagonistic to miR156) target sequences were identified in 2 of the AtqSPL genes (AtqSPL3 and 13) and AtqSPL13 was shown to contain targets for both miR156 and miR172. The expected target sequence for miR172 was identified in AtqAP2 but not in AtqAP2-2 perhaps because the latter sequence is incomplete. Together these results support the model of antagonistic expression of miRNAs 156 and 172 and their target genes during the vegetative to reproductive transition in other species and suggest that this is also the case in *A. tequilana*.

The expression pattern of AtqAP2 correlates with the expected pattern in relation to miR172 suggesting that the AP2/miR172 regulatory model is conserved during the vegetative to reproductive transition in *A. tequilana*. The AtqSPL family can be separated into 2 classes in terms of expression patterns. Closely related genes AtqSPL2 and 8 are expressed at low levels in all tissues tested. AtqSPL9 that is closely related to AtqSPL13 also shows extremely low levels of expression in all tissue tested and may in fact be inactive. In contrast AtqSPL12 and 13 that are found in distinct clades in Online Resource 4 Are strongly expressed in both VSAM and RSAM tissue. However, whereas AtqSPL12 shows higher expression in RSAM compared to VSAM, AtqSPL13 shows decreased expression in RSAM in comparison to VSAM. The expression pattern of AtqSPL13 therefore also coincides with the miR156 expression pattern where miR156 is expressed in VL, RL and RSAM but not in VSAM. The AtqSPL12 expression pattern coincides with miR156 in VSAM but not RSAM. Taken together these results suggest that for AtqSPL13 and possibly AtqSPL12 the SPL/miR156 regulatory model is also conserved during the vegetative to reproductive transition in *A. tequilana*.

### Identification of putative target sequences in *A. tequilana* PGHF32 genes for miRNAS involved in regulation of flowering

**Fig. 9:**
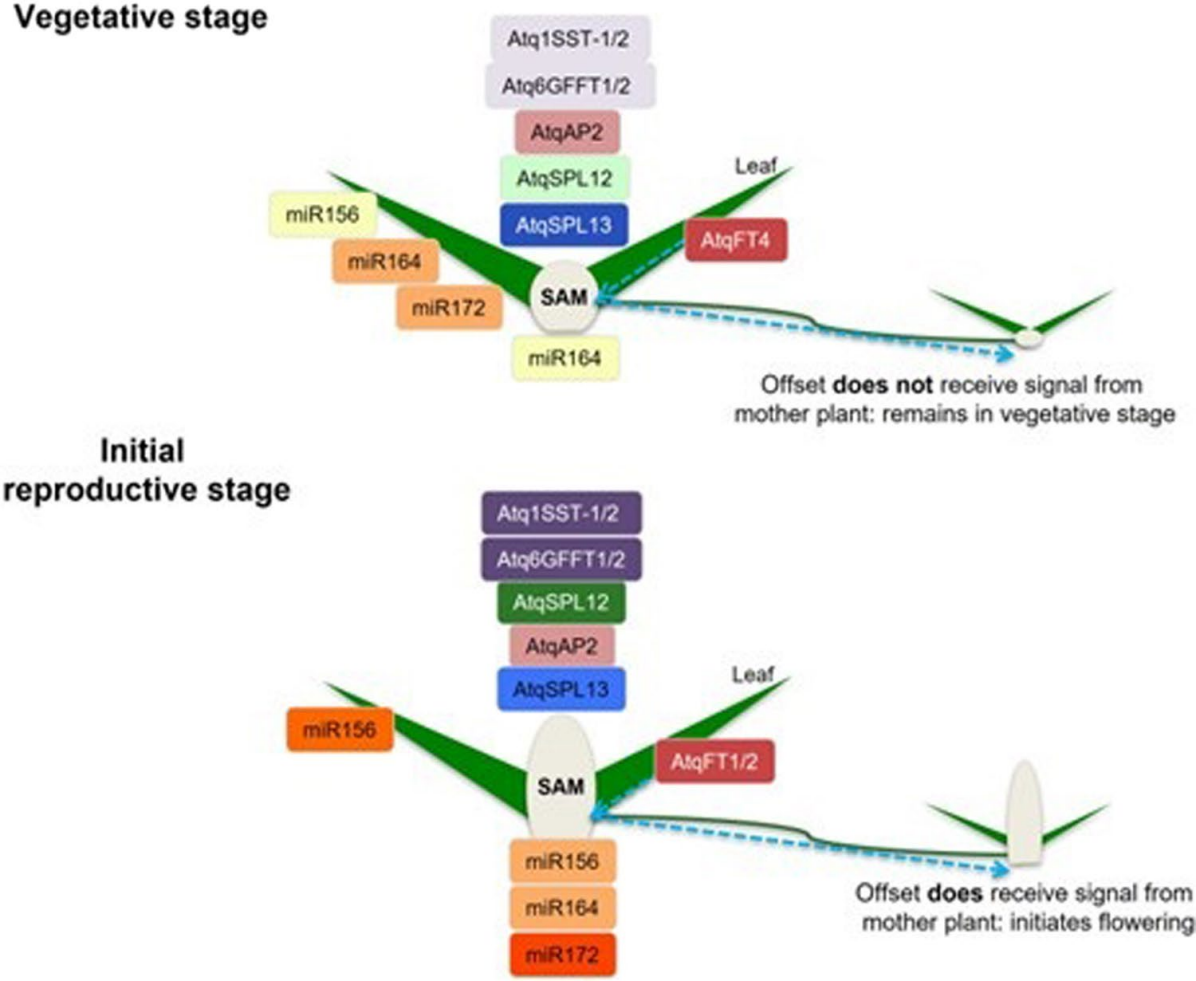
Summary of miRNA and gene expression. The upper diagram shows the expression of miRNAS and target genes in leaf and SAM tissue during the vegetative stage. Intensity of color for each text box indicates level of expression (more intense color = higher expression). The lower diagram shows the expression of miRNAS and target genes in leaf and SAM tissue during the initial reproductive stage. Intensity of color for each text box indicates level of expression (more intense color = higher expression)

Although carbohydrates are known to regulate miR156 (Yang et al. 2013) there are few reports on the occurrence of miR156 or other miRNA target sites in genes involved in carbohydrate metabolism. Since fructan metabolism is fundamental to *Agave* species and the genes encoding enzymes involved in fructan metabolism are expressed differentially during the vegetative to reproductive transition in *A. tequilana* (Ávila de Dios et al. 2019), analysis of PGHF32 family members to identify putative miRNA targets was carried out. Interestingly targets for miR156 but not miR172 were identified in several PGHF32 members. When PGHF32 members of other plant species that are either fructan (onion, asparagus) or non-fructan (*A. thaliana*, sugar beet) producers, were analyzed no targets for miR156 were identified. However targets for miR172 were identified in onion and sugar beet. The other species tested are annual or biennial species, whereas *A. tequilana* is a monocarpic perennial. Although this is a very small sample of different species, this may indicate that at least some members of PGHF32 are subjected to regulation by miR156 in *A. tequilana* and that this regulation may play a role in the vegetative to reproductive transition. Target sequences for miR164 were also found exclusively in the *A. tequilana* PGHF32 genes perhaps also indicating a role for this miRNA in the reproductive process. Notably, the genes encoding enzymes which synthesize fructans (Atq1SST-1 and 2 and Atq6GFFT1 and 2) and additionally AtqSPL13 and AtqSPL12 show regulatory patterns which coincide with their related miRNAs, whereas in general enzymes involved in degradation of fructans or sucrose (AtqVinv1and 2, AtqFEH1 and 3, AtqCwinv2) showed no coincidence in their expression patterns and those of the miRNAS whose targets they harbor. Figure 9 summarizes the results for the AtqFT, SPL, AP2 and PGHF32 genes obtained in this report in addition to their associated miRNAs.

Comparing the results for miRNAs identified in *A. tequilana* PGHF32 with those identified by Ren et al. (2022) to be differentially expressed during flower bud abortion in lotus, miRNAs 156, 164, 396 and 166 are found to be in common, suggesting the possibility of a more general coordinated regulation of these miRNAs during developmental changes.

The results presented confirm the essential role of FT in the vegetative to reproductive transition in *A. tequilana* and suggest that in addition to elements such as AP2, SPL and the miRNA modules previously shown to be involved in the ageing pathway associated with FT regulation in other plant species, PGHF32 in *Agave* may also play an important role. More detailed experimental analysis is needed in order to confirm and extend these observations.

## Conclusions

The results presented confirm the previously proposed functions of AtqFT1 and 2 (activators) and of AtqFT4 (repressor) and show expression of these genes in the schlerenchyma of the vascular tissue of leaves consistent with these functions and with the mobility of FT proteins to the SAM. The AtqFT4 protein shares amino acid changes in the B segment with *B. vulgaris* and *A. cepa*, consistent with repressor function and both the activator and repressor AtqFTs were able to interact with the endogenous components of the *A. thaliana* flowering regulatory apparatus to promote or inhibit flowering. Putative regulatory motifs in the FT promoter regions were identified suggesting roles for light, temperature, hormones and water stress among others in their regulation. AP2 and SPL families and their associated miRNAs are described for the first time for *Agave* species and results suggest conservation of AP2/miR172 and SPL/miR156 regulatory modules during the reproductive transition in *A. tequilana*. Associations between PGHF32 family members and miRNAs 156 and 164 that may be specific to agaves were also identified. Expression patterns for fructan synthesis genes and miR156 and miR164 were shown to coincide in vegetative and reproductive leaf and SAM tissue, supporting a link between carbohydrate metabolism and the regulation of the vegetative to reproductive transition in *A. tequilana*.

### Supplementary Information

Below is the link to the electronic supplementary material.

Supplementary file1 (PDF 972 kb)
