# Supplementary material for: How to awaken a sleeping giant: antagonistic expression of Flowering locus T homologs and elements of the age-related pathway are associated with the flowering transition in Agave tequilana
Source: Plant Reprod. 2023 Dec 12;37(2):111–32. doi: 10.1007/s00497-023-00489-0 (PMC11180032; doi:10.1007/s00497-023-00489-0)
Supplement: Supplementary file 1 — Supplementary file1 (PDF 972 kb) [file 497_2023_489_MOESM1_ESM.pdf]

How to awaken a sleeping giant: Antagonistic Expression of Flowering Locus T Homologs and Elements of the Age Related Pathway are Associated with the Flowering Transition in *Agave tequilana*

Plant Reproduction

Laura Hernández-Soriano[1], Laura Gálvez-Sandre[1], Emmanuel Avila de Dios[1,2] and June Simpson[1]

[1]Department of Genetic Engineering, Cinvestav Unidad Irapuato, Guanajuato, Mexico

[2]Department of Botany and Plant Sciences, University of California, Riverside, California 92521, USA

Correspondence: June Simpson: june.simpson@cinvestav.mx

Online Resource 1 AtqFT primer sequences

Primer	Sequence
FwattB-AtqFT1	GGGGACAAGTTTGTACAAAAAAGCAGGCTTCATGCAAAGAGGGAGGGATCCT
RvattB-AtqFT1	GGGGACCACTTTGTACAAGAAAGCTGGGTCTTAGCTAATCCTTCTTCCTCCGGA
FwattB1-AtqFT2	GGGGACAAGTTTGTACAAAAAAGCAGGCTTCATGAGTAGGGATCCTTTGGT
RvattB2-AtqFT2	GGGGACCACTTTGTACAAGAAAGCTGGGTCTCACAGGTACATCCTTCGCC
FwattB1-AtqFT4	GGGGACAAGTTTGTACAAAAAAGCAGGCTTCATGTCTGCACAATCTTTAGT
RvattB2-AtqFT4	GGGGACCACTTTGTACAAGAAAGCTGGGTCTCAGCCAGCTCTGAACCTTC

Online Resource 2 Cis-element analysis of AtqFT gene promoter regions

AtqFT1	AtqFT2	AtqFT3	AtqFT4	AtqFT5	AtqFT8	AtqFT9	AtqFT10
AACACOREOSGLUB1	10PEHVPSBD	10PEHVPSBD	10PEHVPSBD	ARR1AT	10PEHVPSBD	10PEHVPSBD	ARR1AT
ARR1AT	AACACOREOSGLUB1	ARR1AT	2SSEEDPROTBANAPA	BOXIINTPATPB	ARR1AT	ARR1AT	CACTFTPPCA1
ATHB2ATCONSENSUS	ARR1AT	BOXIINTPATPB	ARR1AT	CACTFTPPCA1	BOXIINTPATPB	BOXIINTPATPB	CANBNNAPA
BOXIINTPATPB	CACTFTPPCA1	CACTFTPPCA1	BOXIINTPATPB	CANBNNAPA	CACTFTPPCA1	CACTFTPPCA1	CBFHV
CACTFTPPCA1	CANBNNAPA	CANBNNAPA	CACTFTPPCA1	CCAATBOX1	CANBNNAPA	CBFHV	CCAATBOX1
CANBNNAPA	CBFHV	CCAATBOX1	CANBNNAPA	CGCGBOXAT	CBFHV	CCAATBOX1	CRTDREHVCBF2
CBFHV	CCAATBOX1	DOFCOREZM	CCAATBOX1	CIACADIANLELHC	CCAATBOX1	DOFCOREZM	DOFCOREZM
CCAATBOX1	CGCGBOXAT	DPBFCOREDCDC3	DOFCOREZM	DOFCOREZM	CIACADIANLELHC	DRE2COREZMRAB17	DRE2COREZMRAB17
CGCGBOXAT	DOFCOREZM	EBOXBNNAPA	DPBFCOREDCDC3	DPBFCOREDCDC3	DOFCOREZM	DRECRTCOREAT	EECCRCAH1
CIACADIANLELHC	DPBFCOREDCDC3	EECCRCAH1	EBOXBNNAPA	EBOXBNNAPA	DPBFCOREDCDC3	EBOXBNNAPA	ELRECOREPCRP1
CRTDREHVCBF2	DRE2COREZMRAB17	ELRECOREPCRP1	EECCRCAH1	EBOXBNNAPA	DRE1COREZMRAB17	EECCRCAH1	GATABOX
DOFCOREZM	DRECRTCOREAT	GAGA8HVBKN3	ELRECOREPCRP1	EECCRCAH1	EBOXBNNAPA	GAGA8HVBKN3	GT1CONSENSUS
DPBFCOREDCDC3	EBOXBNNAPA	GAGAGMGSA1	GAGA8HVBKN3	ELRECOREPCRP1	EECCRCAH1	GAGAGMGSA1	GT1GMSCAM4
DRE1COREZMRAB17	EECCRCAH1	GAREAT	GAGAGMGSA1	GARE1OSREP1	ELRECOREPCRP1	GATABOX	GTGANTG10
DRE2COREZMRAB17	ELRECOREPCRP1	GATABOX	GATABOX	GAREAT	GATABOX	GCCCORE	IBOXCORE
DRECRTCOREAT	GAGA8HVBKN3	GT1CONSENSUS	GCCCORE	GATABOX	GT1CONSENSUS	GCN4OSGLUB1	INRNTPSADB
EBOXBNNAPA	GAGAGMGSA1	GT1GMSCAM4	GCN4OSGLUB1	GT1CONSENSUS	GT1GMSCAM4	GT1CONSENSUS	LTREATLTI78
EECCRCAH1	GARE1OSREP1	GTGANTG10	GT1CONSENSUS	GT1GMSCAM4	GTGANTG10	GT1GMSCAM4	LTRECOREATCOR15
ELRECOREPCRP1	GAREAT	IBOXCORE	GT1GMSCAM4	GTGANTG10	IBOXCORE	GTGANTG10	MYB1AT
GAREAT	GATABOX	INRNTPSADB	GTGANTG10	IBOXCORE	INRNTPSADB	IBOXCORE	MYBCORE
GATABOX	GT1CONSENSUS	MYB1AT	IBOXCORE	INRNTPSADB	LTRE1HVBLT49	INRNTPSADB	MYBST1
GT1CONSENSUS	GT1GMSCAM4	MYBCORE	INRNTPSADB	MYB1AT	MYB1AT	LTRE1HVBLT49	MYCATERD1
GT1GMSCAM4	GTGANTG10	MYBGAHV	LTRE1HVBLT49	MYBCORE	MYB2CONSENSUSAT	LTRECOREATCOR15	MYCATRD22
GTGANTG10	IBOX	MYBPZM	LTRECOREATCOR15	MYBGAHV	MYBCORE	MYB1AT	MYCCONSENSUSAT
IBOXCORE	IBOXCORE	MYBST1	MYB1AT	MYBPLANT	MYBPZM	MYBCORE	NODCON1GM
INRNTPSADB	IBOXCORENT	MYCATERD1	MYB2AT	MYBST1	MYBST1	MYBPZM	NODCON2GM
LTRE1HVBLT49	INRNTPSADB	MYCATRD22	MYB2CONSENSUSAT	MYCATERD1	MYCATERD1	MYBST1	OSE1ROOTNODULE
LTREATLTI78	LTRE1HVBLT49	MYCCONSENSUSAT	MYBCORE	MYCATRD22	MYCATRD22	MYCCONSENSUSAT	OSE2ROOTNODULE
LTRECOREATCOR15	LTRECOREATCOR15	NODCON1GM	MYBCOREATCYCB1	MYCCONSENSUSAT	MYCCONSENSUSAT	NODCON1GM	POLLEN1LELAT52
MYB1AT	MYB1AT	NODCON2GM	MYBPZM	NODCON1GM	NODCON1GM	NODCON2GM	PYRIMIDINEBOXOSRAMY1A
MYBCORE	MYB2CONSENSUSAT	OSE1ROOTNODULE	MYBST1	NODCON2GM	NODCON2GM	OSE1ROOTNODULE	QELEMENTZMZM13
MYBGAHV	MYBCORE	OSE2ROOTNODULE	MYCATERD1	OSE1ROOTNODULE	OSE1ROOTNODULE	OSE2ROOTNODULE	SEF4MOTIFGM7S
MYBPLANT	MYBGAHV	POLLEN1LELAT52	MYCATRD22	OSE2ROOTNODULE	OSE2ROOTNODULE	POLLEN1LELAT52	SORLIP1AT
MYBPZM	MYBPLANT	PRECONSCRHSP70A	MYCCONSENSUSAT	QELEMENTZMZM13	POLLEN1LELAT52	PRECONSCRHSP70A	WBBOXPCWRKY1
MYBST1	MYBPZM	PYRIMIDINEBOXOSRAMY1A	NODCON1GM	RYREPEATLEGUMINBOX	PRECONSCRHSP70A	QELEMENTZMZM13	WBOXATNPR1
MYCATERD1	MYBST1	QELEMENTZMZM13	NODCON2GM	SEBFCONSSTPR10A	PYRIMIDINEBOXOSRAMY1A	RYREPEATLEGUMINBOX	WBOXHVISO1
MYCATRD22	MYCATERD1	RYREPEATLEGUMINBOX	OSE1ROOTNODULE	SEF4MOTIFGM7S	QELEMENTZMZM13	SEF4MOTIFGM7S	WBOXNTCHN48
MYCCONSENSUSAT	MYCATRD22	SEF4MOTIFGM7S	OSE2ROOTNODULE	SORLIP1AT	SEBFCONSSTPR10A	SORLIP1AT	WBOXNTERF3
NODCON1GM	MYCCONSENSUSAT	SORLIP1AT	POLLEN1LELAT52	SORLIP2AT	SORLIP1AT	SORLIP2AT	WRKY71OS
NODCON2GM	NODCON1GM	SORLIP5AT	PRECONSCRHSP70A	TBOXATGAPB	SORLIP2AT	TBOXATGAPB	
OSE1ROOTNODULE	NODCON2GM	TBOXATGAPB	PYRIMIDINEBOXOSRAMY1A	WBBOXPCWRKY1	SORLREP3AT	WBOXHVISO1	
OSE2ROOTNODULE	OSE1ROOTNODULE	WBBOXPCWRKY1	QELEMENTZMZM13	WBOXATNPR1	TBOXATGAPB	WBOXNTCHN48	
PALBOXAPC	OSE2ROOTNODULE	WBOXATNPR1	RYREPEATLEGUMINBOX	WBOXHVISO1	WBOXATNPR1	WBOXNTERF3	
POLLEN1LELAT52	POLLEN1LELAT52	WBOXHVISO1	SEBFCONSSTPR10A	WBOXNTCHN48	WBOXHVISO1	WRKY71OS	
PRECONSCRHSP70A	PRECONSCRHSP70A	WBOXNTCHN48	SEF3MOTIFGM	WBOXNTERF3	WBOXNTCHN48		
PYRIMIDINEBOXOSRAMY1A	PYRIMIDINEBOXOSRAMY1A	WBOXNTERF3	SEF4MOTIFGM7S	WRKY71OS	WBOXNTERF3		
QELEMENTZMZM13	QELEMENTZMZM13	WRKY71OS	SORLIP1AT		WRKY71OS		
SEF4MOTIFGM7S	RYREPEATLEGUMINBOX		SORLIP2AT				
SORLIP1AT	SEBFCONSSTPR10A		SORLIP5AT				
SORLIP2AT	SEF4MOTIFGM7S		SORLREP3AT				
TBOXATGAPB	SORLIP1AT		TBOXATGAPB				
WBBOXPCWRKY1	TBOXATGAPB		WBBOXPCWRKY1				
WBOXATNPR1	WBOXATNPR1		WBOXATNPR1				
WBOXHVISO1	WBOXHVISO1		WBOXHVISO1				
WBOXNTCHN48	WBOXNTCHN48		WBOXNTCHN48				
WBOXNTERF3	WBOXNTERF3		WBOXNTERF3				
WRKY71OS	WRKY71OS		WRKY71OS				

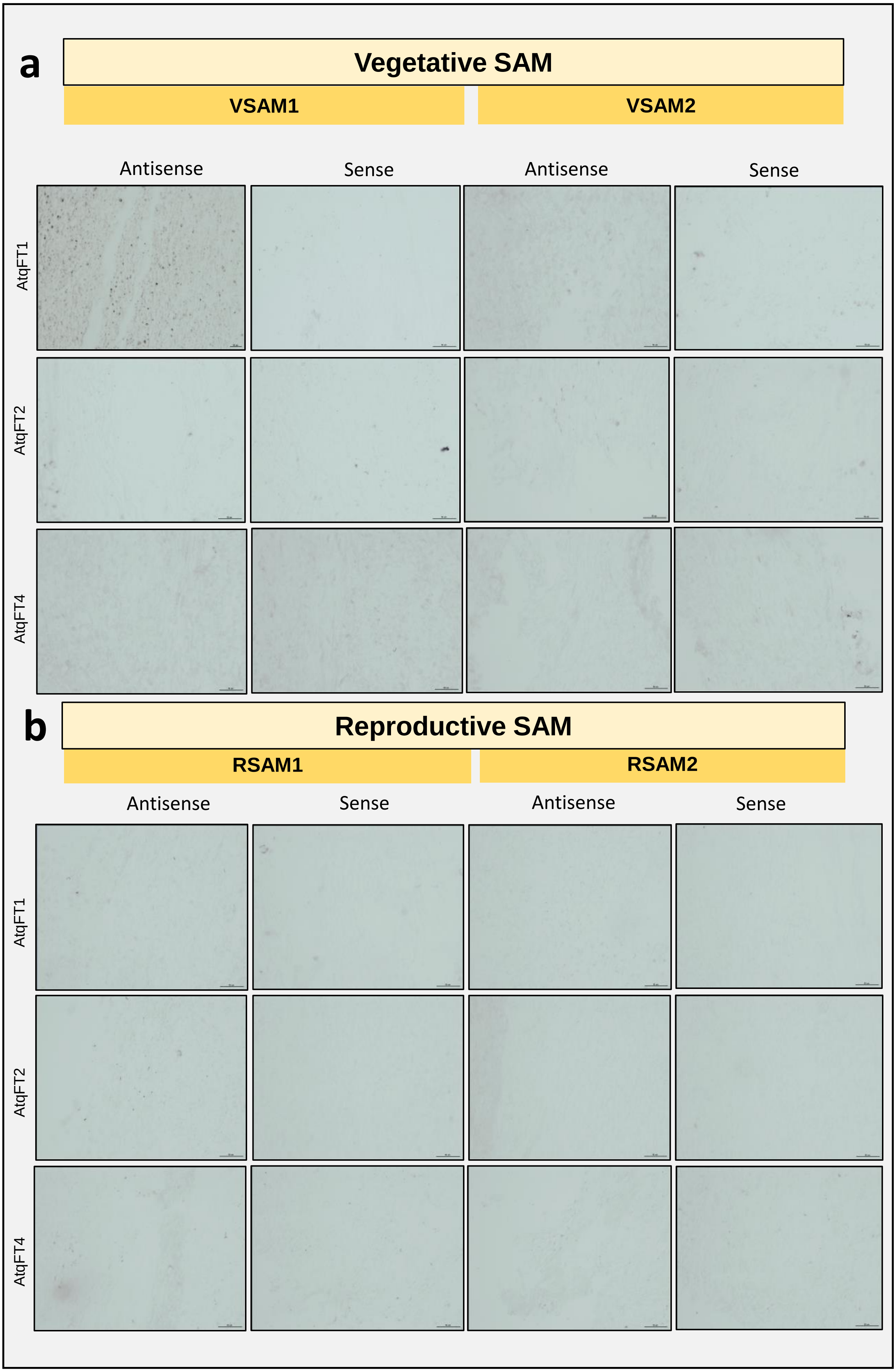
a
Vegetative SAM
VSAM1
VSAM2
Antisense
Sense
Antisense
Sense
AtqFT1
AtqFT2
AtqFT4
b
Reproductive SAM
RSAM1
RSAM2
Antisense
Sense
Antisense
Sense
AtqFT1
AtqFT2
AtqFT4

Online Resource 3 *In situ* hybridization analysis meristem samples. (a) Longitudinal section of vegetative meristem tissues was hybridized with AtqFT1, AtqFT2, and AtqFT4 specific antisense and sense probes. (b) Longitudinal sections of reproductive meristem tissues were hybridized with AtqFT1, AtqFT2, and AtqFT4 specific antisense and sense probes. Bars on *In situ* hybridization analysis are 50 um

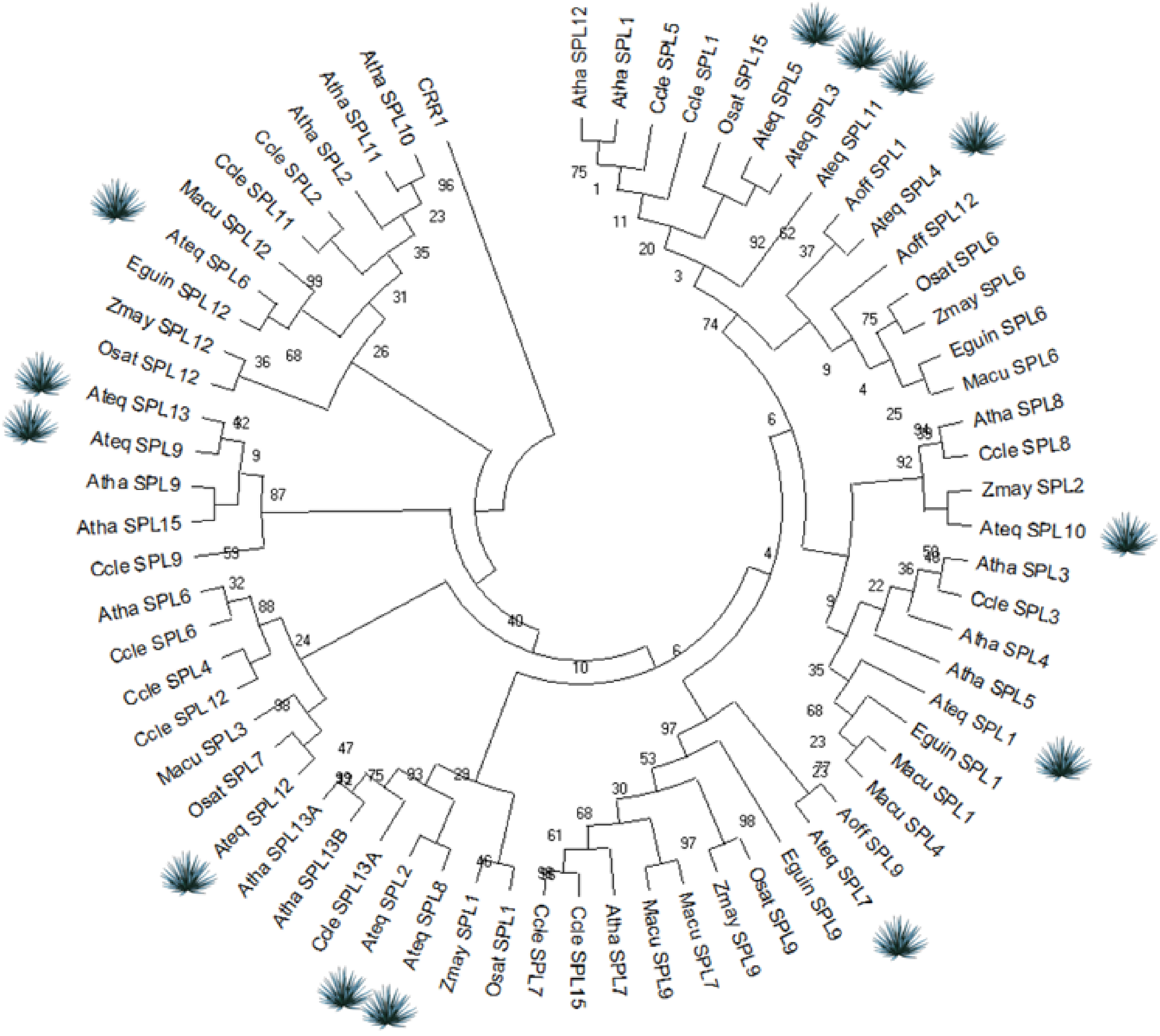

Online Resource 4 Dendrogram showing relationships between *A. tequilana* SPL genes and those of other plant species. Phylogenetic analysis was carried out by using amino acid sequences and considering all sites including gaps/missing information based on the Maximum likelihood method and the JTT+G substitution model. Agave plants indicate AtqSPL genes

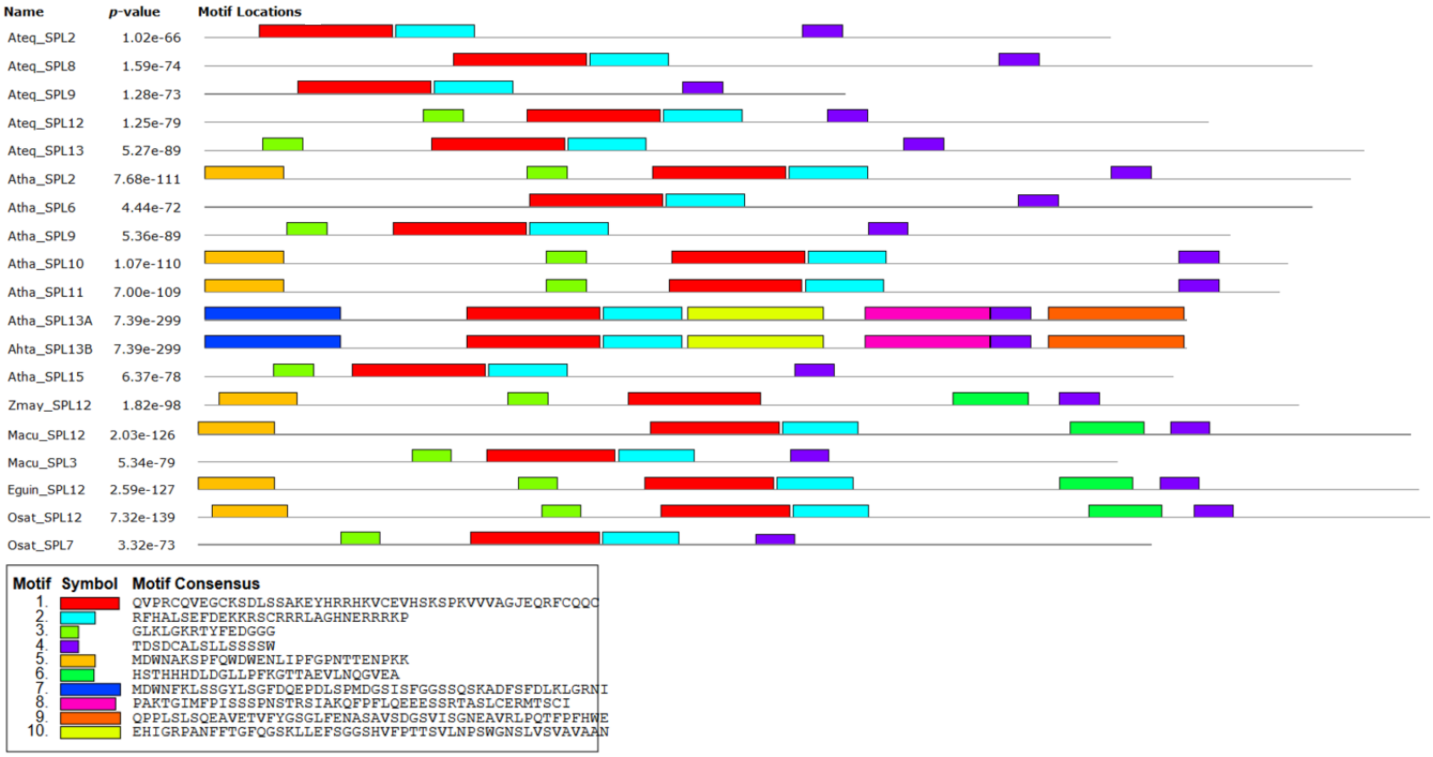

Online Resource 5 Conserved motifs between SPL genes from *A. tequilana* and other plant species. Schematic representation of motif locations in *A. tequilana, A. thaliana, O. sativa, E. guineensis, M. acuminata* and *Z. mays* SPL sequences. Motif enrichment analysis performed with the MEME suite (version 5.4.1)

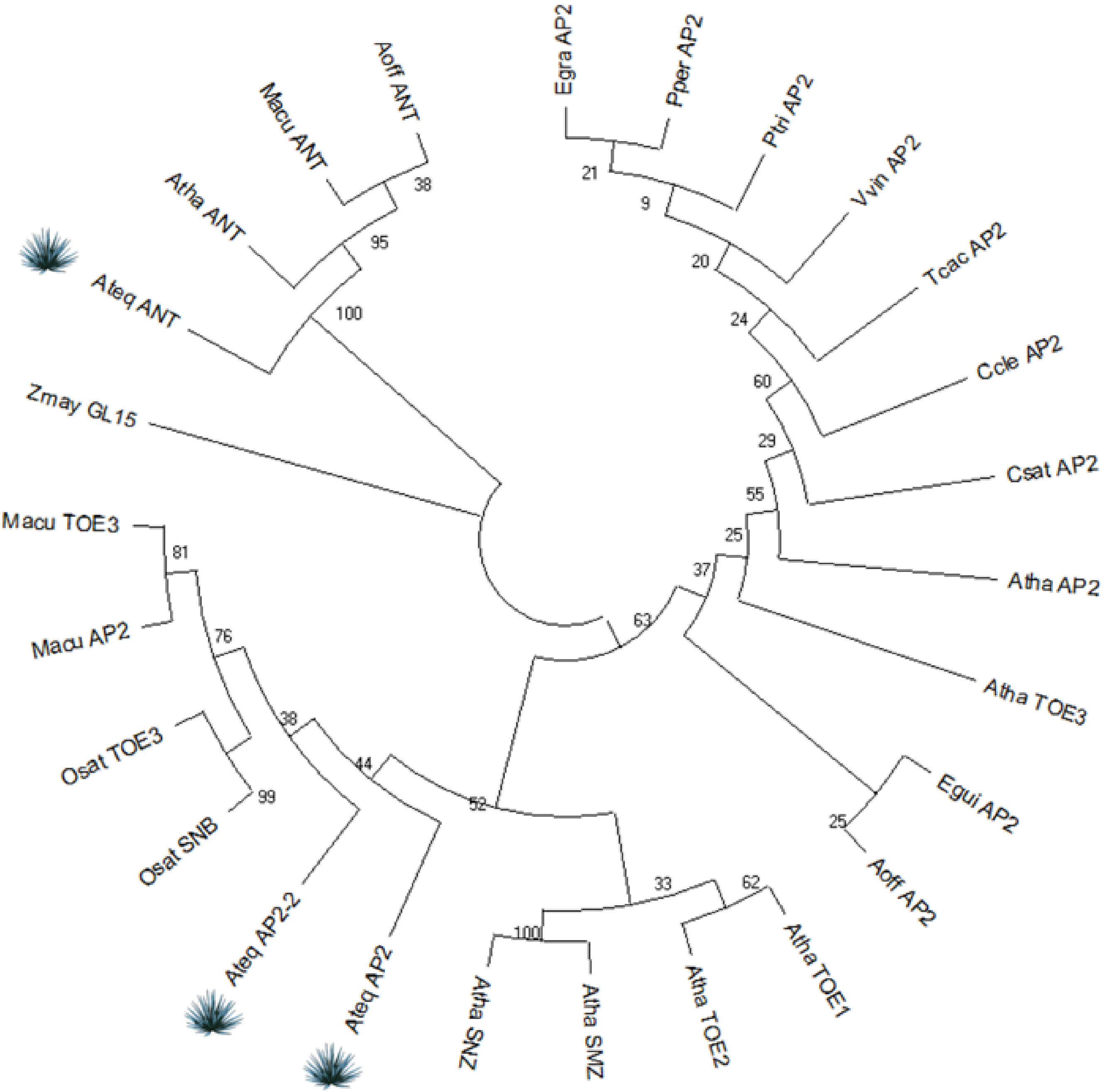

Online Resource 6 Dendrogram showing relationships between *A. tequilana* AP2 genes and those of other plant species. Phylogenetic analysis was carried out by using amino acid sequences and considering all sites including gaps/missing information based on the Maximum likelihood method and the JTT+G substitution model. Agave plant figures indicate AteqAP2 genes

Online Resource 7 miRNA families identified in *A. tequilana* transcriptome using miRbase

miR156	miR167	miR394	miR477	miR845	miR5225
miR157	miR168	miR395	miR479	miR858	miR7528
miR159	miR169	miR396	miR482	miR1515	miR7732
miR160	miR171	miR397	miR528	miR2118	miR7767
miR162	miR172	miR398	miR529	miR3711	miR8175
miR164	miR319	miR399	miR535	miR4414	miR8558
miR165	miR390	miR408	miR827	miR5072	
miR166	miR393	miR444	miR828	miR5083	

Online Resource 8 List of microRNAs targeting AtqSPL and AtqAP2 genes

miRNA target	Gene	miRNA target	Gene
miR156	Ateq_SPL12	miR172	Ateq_AP2
	Ateq_SPL13		Ateq_SPL13
	Ateq_SPL2		Ateq_SPL3
	Ateq_SPL6	miR319	Ateq_SPL11
	Ateq_SPL8		Ateq_SPL3
	Ateq_SPL9		Ateq_SPL5
miR159	Ateq_AP2		Ateq_SPL7
	Ateq_AP2-2		Ateq_SPL8
	Ateq_SPL11	miR396	Ateq_SPL10
	Ateq_SPL3		Ateq_SPL13
	Ateq_SPL4		Ateq_SPL3
	Ateq_SPL5		Ateq_SPL5
miR160	Ateq_SPL12		Ateq_SPL6
miR162	Ateq_SPL4		Ateq_SPL9
miR164	Ateq_SPL4	miR408	Ateq_AP2-2
miR166	Ateq_AP2		Ateq_SPL12
	Ateq_SPL10		Ateq_SPL13
	Ateq_SPL2		Ateq_SPL5
	Ateq_SPL3	miR444	Ateq_SPL12
	Ateq_SPL4	miR482	Ateq_SPL11
	Ateq_SPL5	miR535	Ateq_SPL12
	Ateq_SPL6		Ateq_SPL13
	Ateq_SPL7		Ateq_SPL6
	Ateq_SPL8		Ateq_SPL8
miR167	Ateq_SPL4		Ateq_SPL9
	Ateq_SPL8	miR858	Ateq_SPL4
miR168	Ateq_SPL13	miR1515	Ateq_SPL2
	Ateq_SPL9	miR2118	Ateq_AP2
miR171	Ateq_SPL5	miR2118	Ateq_SPL11
		miR8175	Ateq_SPL10
		miR8558	Ateq_AP2

Online Resource 9 List of microRNAs targeting PGHF32 genes. (a) microRNAs targeting AtqPGHF32 genes. (b) microRNAs targeting PGHF32 genes in *A. tequilana* and other plant species

a

miRNA Target	Gene	miRNA target	Gene	miRNA target	Gene
miR156	Atq1SST-1	miR166	Atq1SST-3	miR396	AtqInv1
	Atq1SST-2		Atq6GFFT-1		AtqInv2
	Atq1SST-3		AtqCwinv-1		AtqVinv1
	AtqCwinv-2		AtqCwinv-2		AtqVinv2
	AtqInv1		AtqFEH-4	miR397	AtqFEH-3
	AtqVinv1		AtqVinv1	miR444	Atq6GFFT-2
	AtqVinv2	miR167	AtqVinv2		AtqVinv2
miR159	AtqFEH-2	miR168	Atq1SST-1	miR477	AtqInv1
	AtqFEH-3		Atq1SST-3	miR479	AtqFEH-4
	AtqVinv1		AtqInv2	miR529	Atq1SST-1
miR164	Atq6GFFT-1		AtqVinv1	miR828	Atq6GFFT-2
	Atq6GFFT-2	miR171	Atq6GFFT-1		AtqFEH-3
	AtqFEH-3	miR319	AtqInv1	miR3711	AtqCwinv-2
	AtqInv1	miR395	AtqInv1	miR5225	Atq1SST-1
miR166	Atq1SST-1	miR396	AtqFEH-1	miR8175	AtqFEH-3
	Atq1SST-2		AtqFEH-3		

b

miRNA target	Gene	miRNA target	Gene	miRNA target	Gene
miR156	Atq1SST-1	miR167	Ac1SST	miR396	Ao1SST
	Atq1SST-2		Ao6GFFT		AoCwinv1-like
	Atq1SST-3		AtqVinv2		AthCwinv2
	AtqCwinv-2		BvVinv		AthCwinv4
	AtqInv1	miR168	Atq1SST-1		BvCwinv
	AtqVinv1		Atq1SST-3		AtqFEH-1
	AtqVinv2		Ao1SST		AtqFEH-3
miR159	AthCwinv1		AthCwinv1		Ao6FEH
	AthCwinv5		AtqInv2		Bv6FEH
	BvCwinv		AtqVinv1		AtqInv1
	AtqFEH-2	miR171	Atq6GFFT-1		AtqInv2
	AtqFEH-3		AoCwinv1-like		AtqVinv1
	Ac1FEH		AthCwinv5		AtqVinv2
	AtqVinv1		Ao6FEH		AthVinv2
miR162	Ao6GFFT	miR172	Ac6GFFT	miR444	Atq6GFFT-2
miR164	Atq6GFFT-1		BvCwinv		AtqVinv2
	Atq6GFFT-2		Ac1FEH	miR477	AtqInv1
	AtqFEH-3	miR319	Ac1SST	miR479	AtqFEH-4
	AtqInv1		Ao1SST	miR529	Atq1SST-1
miR165	Ao1FFT1		Ao6GFFT	miR827	BvVinv
miR166	Atq1SST-1		AthCwinv1	miR828	Atq6GFFT-2
	Atq1SST-2		AthCwinv2		AtqFEH-3
	Atq1SST-3		AthCwinv4	miR1515	Bv6FEH
	Ac1SST		AthCwinv5	miR3711	AtqCwinv-2
	Ao1FFT1		Ac1FEH	miR5072	Ao1FFT1
	Atq6GFFT-1		AtqInv1	miR5225	Atq1SST-1
	Ac6GFFT	miR393	Ac6GFFT	miR8175	AtqFEH-3
	AtqCwinv-1	miR395	Ac1SST		
	AtqCwinv-2		AtqInv1		
	AoCwinv2-like	miR397	AtqFEH-3		
	AthCwinv1	miR398	Ac1SST		
	AtqFEH-4	miR482	AthCwinv4		
	Ath61FEH		Ac1FEH		
	Ath6FEH		BvVinv		
	Bv6FEH	miR8558	Ao1FFT1		
	AtqVinv1		AoCwinv2-like		
	AthVinv2		BvCwinv		
